# Comprehensive Research Synopsis and Systematic Meta-Analyses in Parkinson's Disease Genetics: The PDGene Database

**DOI:** 10.1371/journal.pgen.1002548

**Published:** 2012-03-15

**Authors:** Christina M. Lill, Johannes T. Roehr, Matthew B. McQueen, Fotini K. Kavvoura, Sachin Bagade, Brit-Maren M. Schjeide, Leif M. Schjeide, Esther Meissner, Ute Zauft, Nicole C. Allen, Tian Liu, Marcel Schilling, Kari J. Anderson, Gary Beecham, Daniela Berg, Joanna M. Biernacka, Alexis Brice, Anita L. DeStefano, Chuong B. Do, Nicholas Eriksson, Stewart A. Factor, Matthew J. Farrer, Tatiana Foroud, Thomas Gasser, Taye Hamza, John A. Hardy, Peter Heutink, Erin M. Hill-Burns, Christine Klein, Jeanne C. Latourelle, Demetrius M. Maraganore, Eden R. Martin, Maria Martinez, Richard H. Myers, Michael A. Nalls, Nathan Pankratz, Haydeh Payami, Wataru Satake, William K. Scott, Manu Sharma, Andrew B. Singleton, Kari Stefansson, Tatsushi Toda, Joyce Y. Tung, Jeffery Vance, Nick W. Wood, Cyrus P. Zabetian, 23andMe, The Genetic Epidemiology of Parkinson's Disease (GEO-PD) Consortium, The International Parkinson's Disease Genomics Consortium (IPDGC), The Parkinson's Disease GWAS Consortium, The Wellcome Trust Case Control Consortium 2 (WTCCC2), Peter Young, Rudolph E. Tanzi, Muin J. Khoury, Frauke Zipp, Hans Lehrach, John P. A. Ioannidis, Lars Bertram

**Affiliations:** 1 Neuropsychiatric Genetics Group, Department of Vertebrate Genomics, Max Planck Institute for Molecular Genetics, Berlin, Germany; 2 Department of Neurology, Massachusetts General Hospital, Charlestown, Massachusetts, United States of America; 3 Department of Neurology, Medical Center of the Johannes Gutenberg-University, Mainz, Germany; 4 Department of Neurology, University Hospital, Münster, Germany; 5 Department of Mathematics and Computer Science, Free University, Berlin, Germany; 6 Institute for Behavioral Genetics, University of Colorado, Boulder, Colorado, United States of America; 7 Clinical and Molecular Epidemiology Unit, Department of Hygiene and Epidemiology, University of Ioannina School of Medicine, Ioannina, Greece; 8 Centre for Diabetes and Endocrinology, Royal Berkshire Hospital, Reading, United Kingdom; 9 Oxford Centre for Diabetes, Endocrinology, and Metabolism, Churchill Hospital, University of Oxford, Oxford, United Kingdom; 10 Max Planck Institute for Human Development, Berlin, Germany; 11 Department of Health Sciences Research, Mayo Clinic, Rochester, Minnesota, United States of America; 12 John P. Hussman Institute for Human Genomics, Miller School of Medicine, University of Miami, Miami, Florida, United States of America; 13 Department for Neurodegenerative Diseases, Hertie Institute for Clinical Brain Research, University of Tübingen, Tübingen, Germany; 14 DZNE, German Center for Neurodegenerative Diseases, Tübingen, Germany; 15 INSERM, UMR_S975, Paris, France; 16 Université Pierre et Marie Curie-Paris, Centre de Recherche de l'Institut du Cerveau et de la Moelle épinière, UMR-S975, Paris, France; 17 CNRS, UMR 7225, Paris, France; 18 AP-HP, Pitié-Salpêtrière Hospital, Department of Genetics and Cytogenetics, Paris, France; 19 Department of Neurology, Boston University School of Medicine, Boston University, Boston, Massachusetts, United States of America; 20 Department of Biostatistics, Boston University School of Public Health, Boston, Massachusetts, United States of America; 21 23andMe, Mountain View, California, United States of America; 22 Department of Neurology, Emory University School of Medicine, Atlanta, Georgia, United States of America; 23 Department of Medical Genetics, University of British Columbia, Vancouver, Canada; 24 Indiana University School of Medicine, Indianapolis, Indiana, United States of America; 25 New York State Department of Health Wadsworth Center, Albany, New York, United States of America; 26 Department of Molecular Neuroscience, UCL Institute of Neurology, University College London, London, United Kingdom; 27 Department of Clinical Genetics, Section of Medical Genomics, VU University Medical Centre, Amsterdam, The Netherlands; 28 Section of Clinical and Molecular Neurogenetics, Department of Neurology, University of Lübeck, Lübeck, Germany; 29 Department of Neurology, NorthShore University Health System, Evanston, Illinois, United States of America; 30 INSERM UMR 1043, CPTP, Toulouse, France; 31 Paul Sabatier University, Toulouse, France; 32 Laboratory of Neurogenetics, National Institute on Aging, National Institutes of Health, Bethesda, Maryland, United States of America; 33 Division of Neurology/Molecular Brain Science, Kobe University Graduate School of Medicine, Kobe, Japan; 34 deCODE genetics, Reykjavik, Iceland; 35 UCL Genetics Institute, University College London, London, United Kingdom; 36 Department of Molecular Neuroscience, UCL Institute of Neurology, University College London, London, United Kingdom; 37 VA Puget Sound Health Care System and Department of Neurology, University of Washington, Seattle, Washington, United States of America; 38 Office of Public Health Genomics, Centers for Disease Control and Prevention, Atlanta, Georgia, United States of America; 39 Biomedical Research Institute, Foundation for Research and Technology–Hellas, Ioannina, Greece; 40 Center for Genetic Epidemiology and Modeling and Tufts Clinical and Translational Science Institute, Tufts University School of Medicine, Boston, Massachusetts, United States of America; 41 Stanford Prevention Research Center, Department of Medicine and Department of Health Research and Policy, Stanford University School of Medicine, Stanford, California, United States of America; University of Miami, United States of America

## Abstract

More than 800 published genetic association studies have implicated dozens of potential risk loci in Parkinson's disease (PD). To facilitate the interpretation of these findings, we have created a dedicated online resource, PDGene, that comprehensively collects and meta-analyzes all published studies in the field. A systematic literature screen of ∼27,000 articles yielded 828 eligible articles from which relevant data were extracted. In addition, individual-level data from three publicly available genome-wide association studies (GWAS) were obtained and subjected to genotype imputation and analysis. Overall, we performed meta-analyses on more than seven million polymorphisms originating either from GWAS datasets and/or from smaller scale PD association studies. Meta-analyses on 147 SNPs were supplemented by unpublished GWAS data from up to 16,452 PD cases and 48,810 controls. Eleven loci showed genome-wide significant (*P*<5×10^−8^) association with disease risk: *BST1*, *CCDC62/HIP1R*, *DGKQ/GAK*, *GBA*, *LRRK2*, *MAPT*, *MCCC1/LAMP3*, PARK16, *SNCA*, *STK39*, and *SYT11/RAB25*. In addition, we identified novel evidence for genome-wide significant association with a polymorphism in *ITGA8* (rs7077361, OR 0.88, *P* = 1.3×10^−8^). All meta-analysis results are freely available on a dedicated online database (www.pdgene.org), which is cross-linked with a customized track on the UCSC Genome Browser. Our study provides an exhaustive and up-to-date summary of the status of PD genetics research that can be readily scaled to include the results of future large-scale genetics projects, including next-generation sequencing studies.

## Author Summary

The genetic basis of Parkinson's disease is complex, i.e. it is determined by a number of different disease-causing and disease-predisposing genes. Especially the latter have proven difficult to find, evidenced by more than 800 published genetic association studies, typically showing discrepant results. To facilitate the interpretation of this large and continuously increasing body of data, we have created a freely available online database (“PDGene”: http://www.pdgene.org) which provides an exhaustive account of all published genetic association studies in PD. One particularly useful feature is the calculation and display of up-to-date summary statistics of published data for overlapping DNA sequence variants (polymorphisms). These meta-analyses revealed eleven gene loci that showed a statistically very significant (*P*<5×10^−8^; a.k.a. genome-wide significance) association with risk for PD: *BST1*, *CCDC62/HIP1R*, *DGKQ/GAK*, *GBA*, *LRRK2*, *MAPT*, *MCCC1/LAMP3*, PARK16, *SNCA*, *STK39*, *SYT11/RAB25*. In addition and purely by data-mining, we identified one novel PD susceptibility locus in a gene called *ITGA8* (rs7077361, *P* = 1.3×10^−8^). We note that our continuously updated database represents the most comprehensive research synopsis of genetic association studies in PD to date. In addition to vastly facilitating the work of other PD geneticists, our approach may serve as a valuable example for other complex diseases.

## Introduction

Parkinson's disease (PD) is the second most common neurodegenerative disease with a prevalence of ∼1% over 60 years of age [1]. Approximately 5–10% of the patients show an autosomal dominant or recessive mode of inheritance, and several causative genes have been identified, e.g. *SNCA*, *LRRK2*, *PARK2*, and *PINK1* (for review see ref. [2]). Recently, two other novel autosomal dominant PD genes, *VPS35* and *EIF4G1* [3]–[5], have been identified, the former via application of next-generation sequencing techniques. It can be anticipated that causal mutations in additional genes will emerge within the next years. However, the vast majority of patients suffer from non-Mendelian forms of PD, which are likely caused by the combined effects of genetic and environmental factors. In order to decipher the genetic architecture underlying PD susceptibility, more than 800 genetic association studies have been performed over the past 20 years. While early candidate gene studies and subsequent meta-analyses provided conclusive evidence showing that polymorphisms in *SNCA* [6] (encoding alpha-synuclein), *LRRK2* [7] (leucine-rich repeat kinase 2), *MAPT* [8] (microtubule-associated protein tau), and *GBA* [9] (acid beta-glucosidase) significantly impact PD susceptibility, most association studies in the field provided inconclusive or even conflicting results.

During the last few years, genome-wide association studies (GWAS) [10]–[19] have postulated additional PD loci. While the early GWAS and a GWAS-meta-analysis [20] were of limited sample sizes and yielded mostly inconsistent results, more recent studies have identified a number of loci that were independently confirmed in follow-up studies (e.g. *GAK*, *BST1*, and PARK16, see Table 1 for all proposed GWAS findings across GWAS publications). Very recently, a GWAS meta-analysis [21] implicated several other new putative PD loci which currently await further validation. Despite this progress, approximately 40% or more of the population-attributable risk probably remains unexplained by today's most promising PD loci [21]. To this end, genetic association studies remain one of the mainstays of PD genetics research. However, GWAS and other large-scale association studies typically only highlight the most promising results and often do not provide data on variants showing suggestive evidence for association, or previously implied variants that could not be confirmed in the GWAS setting. As a result, the cumulative genetic evidence in favor of or against association with certain variants in the PD field is becoming increasingly difficult to follow, evaluate and interpret. To address this problem, we have comprehensively collected, catalogued and systematically meta-analyzed the data from all genetic association studies published in the field of non-Mendelian PD, including GWAS, and made all results publicly available on a regularly updated online database, “PDGene” (http://www.pdgene.org).

**Table 1:** Overview of genome-wide association studies (GWAS) published in PD until March 31, 2011.

GWAS	Design GWAS (Follow-up)	Population GWAS (Follow-up)	# SNPs	# PD GWAS (Follow-up)	# CTRL GWAS (Follow-up)	“Featured” genetic loci
Maraganore, 2005 (ref. 9)	Family-based (case-control)	USA-LEAPS (USA)	198,345	443 (332)	443 (332)	*CDCP2*, *GALNT3*, *GWA_2q36.3*, *GWA_4q28.1*, *GWA_4q28.3*, *GWA_5p15.32*, *GWA_7p14.2*, *GWA_10q21.1*, *PASD1*, *PRDM2*, *SEMA5A*
Fung, 2006 (ref. 10)	Case-control (-)	USA-NINDS	408,803	267 (-)	270 (-)	*BRDG*, *DLG2*, *GLT25D2*, *GWA_4q35.2*, *GWA_7p12*, *GWA_10q11.21*, *GWA_11q11*, *GWA_16q23.1*, *GWA_22q13*, *NEGR1*, *ULK2*, *ZNF313*
Pankratz, 2009 (ref. 11)	Case-control (-)	USA-PROGENI/GenePD (-)	328,189	857 (-)	867 (-)	***DGKQ/GAK***, *GPRIN3*, ***MAPT***, ***SNCA***
Simon-Sanchez, 2009 (ref. 12)	Case-control (case-control)	USA-NINDS, Germany(USA, Germany, UK)	463,185	1,745 (3,452)	4,047 (4,756)	***LRRK2***, ***MAPT***, **PARK16**, ***SNCA***
Satake, 2009 (ref. 13)	Case-control (case-control)	Japan (Japan)	435,470	1,078 (993)	2,628 (15,753)	***BST1***, ***LRRK2***, **PARK16**, ***SNCA***
Edwards, 2010 (ref. 14)	Case-control (-)	USA-HIHG (-)	491,376	604 (-)	619 (-)	***MAPT***, ***SNCA***
Hamza, 2010 (ref. 15)	Case-control (-)	USA-NGRC (-)	811,597	2,000 (-)	1,986 (-)	***GAK/DGKQ*** *, HLA* locus, ***MAPT***, ***SNCA***
Spencer, 2011 (ref. 16)	Case-control (case-control)	UK-WTCCC2 (France)	1,733,533	1,705 (1,039)	5,175 (1,984)	***BST1*** **, ** ***GAK/DGKQ*** **, ** ***MAPT*** **, PARK16, ** ***SNCA***
Saad, 2011 (ref. 17)	Case-control (case-control)	France (UK-WTCCC2, Australia)	492,929	1,039 (3,232)	1,984 (7,064)	***BST1*** *, GWA_12q24, * ***SNCA***
Simon-Sanchez, 2011 (ref. 18)	Case-control (case-control)	Netherlands	514,799	772 (-)	2024 (-)	***BST1***, *HLA* locus, ***GAK/DGKQ*** **, ** ***MAPT*** **, ** ***SNCA***

The overview is based on content on the PDGene website (http://www.pdgene.org; current on March 31^st^, 2011). Studies are listed in order of publication date. ‘# PD GWAS’ and ‘# CTRL GWAS’ refers to sample sizes used in the initial GWAS datasets, whereas ‘Follow-up’ refers to the total number of replication samples where applicable. ‘Featured genes’ are those genes/loci that were declared as ‘associated’ in the original publication; note that criteria for declaring association varies across studies. Genetic loci in bold font denote genes showing genome-wide significant results (*P*<5×10^−8^) in the PDGene meta-analyses.

## Results

### Database content

The results of this research synopsis are based on a freeze of the PDGene database content on March 31^st^ 2011 (available upon request from the authors). At that time, PDGene included details on 828 individual studies across more than 50 different countries and six continents reporting on 3,382 polymorphisms in 890 genetic loci. Data for more than 2,000 SNPs were supplemented by results derived from up to three publicly available GWAS datasets [10], [12], [13] following extensive quality control and imputation. Ultimately, this procedure yielded a total of 867 polymorphisms across ∼300 genetic loci that met our criteria for meta-analysis (see Methods). Additional independent GWAS data for 147 SNPs yielding *P* values of ≤0.1 in these initial meta-analyses were provided by researchers of all remaining currently published Caucasian GWAS datasets [13], [15]–[19], [22]. Following the identification of genome-wide significant association with an intronic SNP (rs7077361) in *ITGA8* after addition of these data, we obtained additional data from the same GWAS datasets on ∼1,400 SNPs in the chromosomal region encompassing *ITGA8* (chr10:15346353–15801533, hg18). Finally, independent replication data in Caucasian and Asian populations from the GEO-PD consortium [23] generated for ten recently described PD loci [21] were made available for inclusion. As a result, we were able to substantially increase the sample size (up to 16,452 PD cases and 48,810 controls) for a large number of some of the most promising PD loci. For instance, we were able to add data from up to 48,861 previously not analyzed combined cases and controls to meta-analyses of some of the recently proposed PD loci [21] (median sample size 14,896, see Table 2 and Table S1 for details). In addition to these focused analyses, PDGene displays meta-analysis results for more than seven million additional SNPs originating from up to three publicly available GWAS datasets [10], [12], [13]. The results are available online (e.g. as summarized in http://www.pdgene.org/largescalemeta.asp), where they are cross-linked to a customized and fully browsable track on the UCSC Genome Browser.

**Table 2:** Genome-wide significant summary meta-analysis results of the PDGene database in populations of Caucasian and Asian decent.

Caucasian ethnicity											
Locus	Polymorphism	Location (hg18)	MAF	Allele contrast	N datasets	N samples	OR (95% CI)	*P*-value	*I* ^2^ (95% CI)	HuGENet	BF
*GBA*	N370S	chr1:153451576	0.01	G vs. A	15	44,851	3.51 (2.55–4.83)	1.44×10^−14^	38 (0–66)	A	6.6
*SYT11/RAB25*	chr1:154105678	chr1:154105678	0.02	T vs. C	6	17,300	1.73 (1.48–2.02)	2.35×10^−12^	0 (0–52)	B*	8.2
PARK16	rs947211	chr1:204019288	0.23	A vs. G	12	69,262	0.91 (0.88–0.94)	8.00×10^−10^	0 (0–66)	A	6.8
*STK39*	rs2390669	chr2:168800188	0.13	C vs. A	14	35,159	1.19 (1.12–1.25)	1.37×10^−09^	18 (0–56)	A	4.9*
*MCCC1/LAMP3*	rs11711441	chr3:184303969	0.14	A vs. G	25	46,502	0.86 (0.82–0.91)	9.20×10^−10^	18 (0–50)	A	6.8
*DGKQ*	rs11248060	chr4:954359	0.12	T vs. C	10	57,716	1.21 (1.15–1.27)	3.04×10^−12^	11 (0–52)	A	9.2
*BST1*	rs11724635	chr4:15346199	0.43	C vs. A	26	46,586	0.88 (0.84–0.91)	1.87×10^−10^	43 (10–64)	A	7.5
*SNCA*	rs356219	chr4:90856624	0.41	G vs. A	31	79,494	1.29 (1.25–1.33)	6.06×10^−65^	16 (0–46)	A	61.0
*ITGA8*	rs7077361	chr10:15601549	0.12	C vs. T	11	61,036	0.88 (0.84–0.92)	1.51×10^−08^	0 (0–55)	A	5.7
*LRRK2*	rs1491942	chr12:38907075	0.21	G vs. C	21	34,123	1.17 (1.13–1.22)	6.44×10^−15^	0 (0–38)	A	11.8
*CCDC62/HIP1R*	rs10847864	chr12:121892551	0.39	T vs. G	23	38,367	1.15 (1.11–1.18)	4.37×10^−17^	0 (0–35)	A	14.4
*MAPT/STH*	H1H2	chr17:42131818–41149582	0.20	H2 vs. H1	37	50,389	0.78 (0.75–0.80)	7.97×10^−52^	0 (0–29)	A	48.1

Asian ethnicity											
Locus	Polymorphism	Location (hg18)	MAF	Allele contrast	N datasets	N samples	OR (95% CI)	*P*-value	*I* ^2^ (95% CI)	HuGENet	BF
PARK16	rs823156	chr1:204031263	0.17	G vs. A	5	22,870	0.74 (0.68–0.81)	2.09×10^−12^	0 (0–58)	A	9.2
*BST1*	rs4538475	chr4:15347035	0.38	G vs. A	3	20,393	0.80 (0.75–0.86)	9.53×10^−10^	0 (-)	A	6.8
*SNCA*	rs6532194	chr4:90999925	0.40	T vs. C	5	22,844	1.29 (1.20–1.39)	4.91×10^−11^	31 (0–74)	A	8.0
*LRRK2*	rs34778348	chr12:39043595	0.04	A vs. G	13	10,441	2.23 (1.89–2.63)	2.97×10^−21^	0 (0–53)	B*	15.2

Whenever multiple polymorphisms showed genome-wide significant association in the same locus, only the variant with the smallest *P*-value is listed here. Note that, overall, 103 PDGene meta-analyses results across the 12 loci listed above yield genome-wide significant evidence for association with PD. For a complete list of these as well as the non-genome-wide significant meta-analysis results performed for the datafreeze, see Table S1. MAF = minor allele frequency in cases and controls combined; N = Number, OR = Odds Ratio; CI = confidence interval; *I* ^2^ = estimate of percentage of between-study heterogeneity that is beyond chance. BF = Bayes factor. *Note that additional polymorphisms in these loci showing genome-wide significant association with PD yield are graded with “strong epidemiologic credibility” (grade A) according to the HuGENet criteria [26], [27], and a Bayes Factor >5 [25], respectively (see Table S1 for more details).

### PDGene meta-analysis results

**Figure 1:**
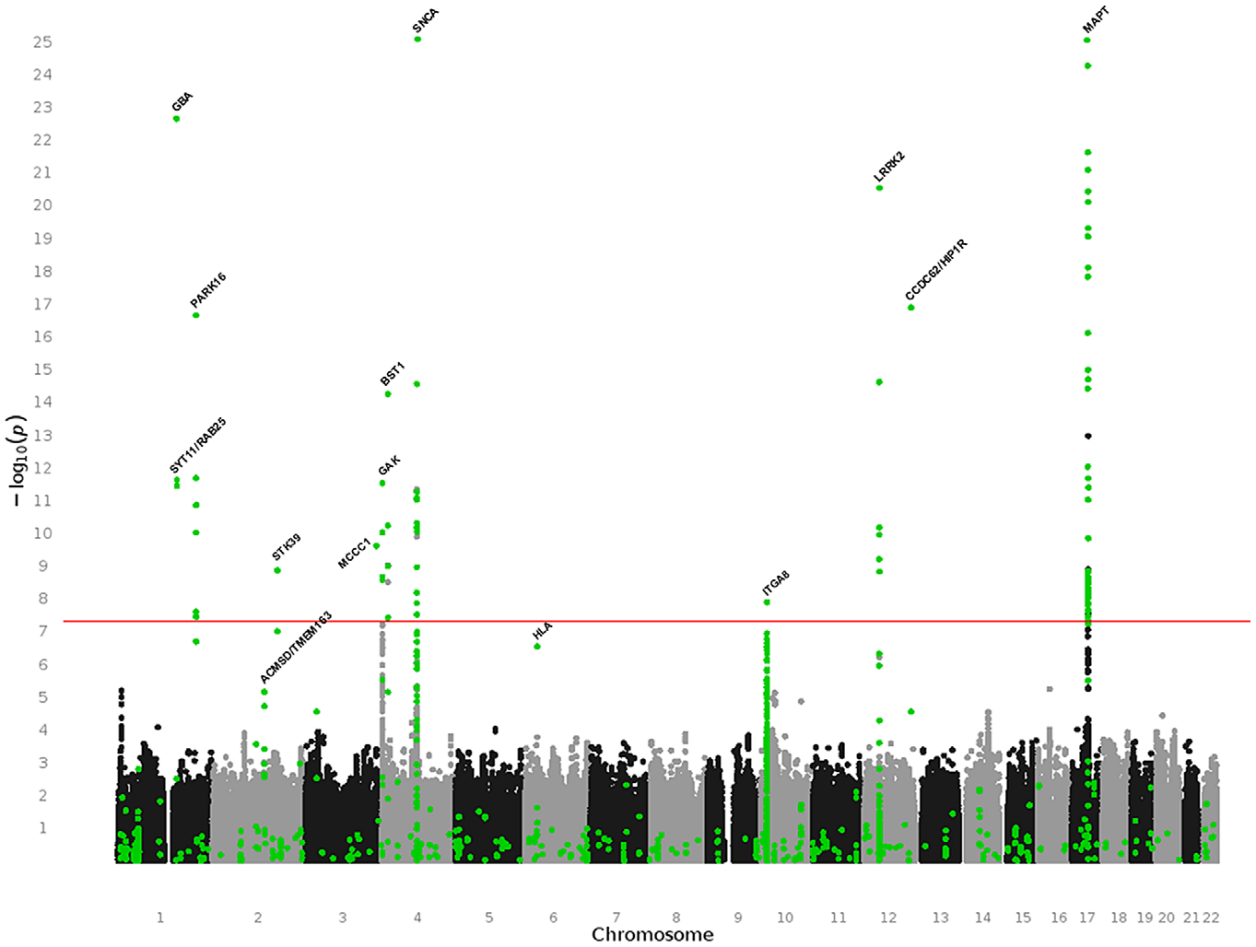
Manhattan plot of all meta-analysis results performed in PDGene. This summary combines association results from 7,123,986 random-effects meta-analyses based on the March 31^st^ 2011 datafreeze of the PDGene database. Results are plotted as −log_10_ *P*-values (y-axis) against physical chromosomal location (x-axis). Black and grey dots indicate results originating exclusively from the three fully publicly available GWAS datasets [10], [12], [13] (see Methods), while green dots are based on a combination of smaller scale studies, supplemented by GWAS datasets (where applicable). Gene annotations are provided for genes highlighted in the main text.

The PDGene meta-analyses of the 867 core polymorphisms were based on a median of 7,680 subjects (interquartile range 4,612–16,726). Additional meta-analyses were performed after stratification for Caucasian and Asian ancestry (for details on sample size and included ethnicities for individual meta-analyses see Table S1). In addition, we also performed random-effects meta-analyses across all three publicly available GWAS datasets [10], [12], [13] following genotype imputation using data from the International HapMap Consortium and 1000 Genomes Project. Ultimately this yielded 7,123,920 SNPs that could be meta-analyzed across at least two GWAS datasets (see Figure S1 for a quantile-to-quantile plot of the GWAS-only meta-analyses). All 867 core meta-analysis results are available online on PDGene as forest plots, summarizing the relative contributions of each dataset to the most current summary effect estimate, and in the form of cumulative plots, illustrating how summary ORs evolve over time. All meta-analysis results are plotted in Figure 1 (green dots) alongside the GWAS-only meta-analysis results (black and grey dots).

One-hundred-three meta-analyses across 12 genetic loci (*BST1*, *CCDC62/HIP1R*, *DGKQ/GAK*, *GBA*, *ITGA8*, *LRRK2*, *MAPT*, *MCCC1/LAMP3*, PARK16, *SNCA*, *STK39*, *SYT11/RAB25*) yielded summary ORs suggesting a genome-wide significant (*P*≤5×10^−8^) increase or decrease in PD risk in all ethnicities and/or after stratification for ethnic ancestry (Table 2, Table S1, and Figure S2 [forest plots]). None of these loci contained more than one SNP independently associated at genome-wide significance (as judged by pair-wise linkage disequilibrium assessments using ‘SNAP’ and r^2^-values of 0.2 as cut off http://www.broadinstitute.org/mpg/snap/). The majority of polymorphisms tested in the genome-wide significant loci do not show evidence for publication bias (Table S1). Finally, all genome-wide significant signals were robust against potential undetected sample overlap using a recently proposed procedure [24] (see Table S2 for more details). Combined sample sizes for all 12 loci were substantially larger here as compared to any previously published meta-analysis (Table S1), providing unequivocal evidence for an involvement of these loci in PD susceptibility. While power to detect genome-wide significance was excellent for most of these loci (>80% based on an OR of 1.15, and a minor allele frequency down to 0.05 using the Genetic Power Calculator, http://pngu.mgh.harvard.edu/~purcell/gpc/), power was less for a large number of other meta-analyses due to smaller sample sizes and allele frequencies (see Table S1 for details). Thus, no simple statistic can summarize the overall power of our study.

**Figure 2:**
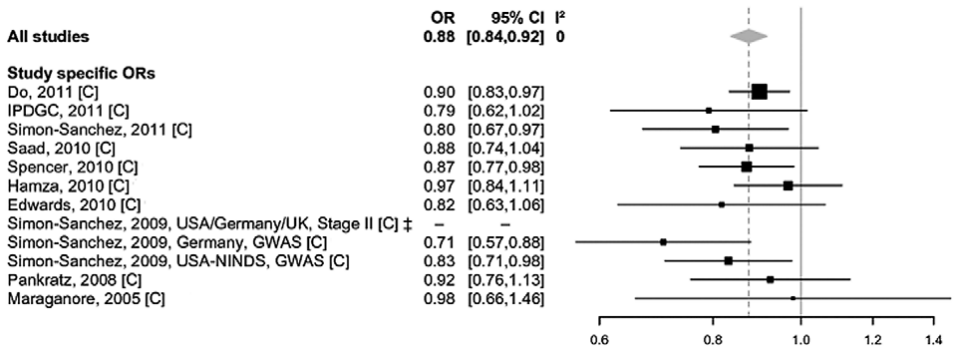
Forest plot of the meta-analysis of rs7077361 in *ITGA8*. Study-specific allelic odds ratios (ORs, black squares) and 95% confidence intervals (CIs, lines) were calculated for each included dataset. The summary OR and CI was calculated using the DerSimonian Laird random-effects model (grey diamond) [31]. C = Caucasian ancestry.

The above list includes an intronic polymorphism in *ITGA8* located on chromosome 10p13 for which we identified novel evidence for genome-wide association with PD risk (OR 0.88, *P* = 1.3×10^−8^, *I* ^2^ = 0, see Table 2, and Figure 2). This SNP had previously been proposed to be associated with PD risk at sub-genome-wide significance by Simon-Sanchez et al [13]. After obtaining and meta-analyzing GWAS data from ∼1,400 additional SNPs in this region derived from all Caucasians GWAS datasets [10], [12], [13], [15]–[19], [21], [22], rs7077361 remained the most significantly associated SNP in this region (Figure S3).

In addition to using random-effects models, we also performed exploratory fixed-effect meta-analyses on all eligible polymorphisms. These analyses did not reveal genome-wide significant effect sizes for any additional locus, except *ACMSD/TMEM163* (most significant SNP rs6723108, OR 0.91, *P* = 1.3×10^−9^, *I* ^2^ = 46% [95% CI 0–73%], Figure S4, panel 1) and *HLA* (most significant SNP chr6:32609909, OR 0.78, *P* = 8.8×10^−15^, *I* ^2^ = 84% [95% CI 70–91%], Figure S4, panel 2), both of which were reported to be associated with PD risk at genome-wide significance in previous work [16], [21]. In both instances, the lack of genome-wide significance in the random-effects models (Table S1) was due to relatively pronounced heterogeneity of effect estimates across studies. However, the heterogeneity across the 11 datasets in the *ACMSD/TMEM163* meta-analysis is almost entirely due to variance of effect size estimates in the same direction (see Figure S4, panel 1), making it likely that *ACMSD/TMEM163* represents a genuine PD risk locus. For the SNP tested in the *HLA* locus (chr6:32609909, Figure S4, panel 2), heterogeneity is more pronounced and more complex owing to ORs on either side of 1. This could be due to a number of reasons, e.g. subtle and uncorrected population substructure and/or different LD patterns between the analyzed SNP and the actual functional variant(s) [16]. Thus, although the evidence is currently not as conclusive as for *ACMSD/TMEM163* it still appears quite possible that there is one or more PD association signals in the *HLA* region. Regardless of these considerations, additional data are needed to more firmly assess the role of both loci in contributing to PD susceptibility.

### Ethnicity-specific meta-analysis results

*SNCA*, *LRRK2*, *BST1*, and PARK16 show evidence for genome-wide significance in meta-analyses restricted to Caucasian and Asian populations (Table 2). Furthermore, data obtained from the GEO-PD consortium [23] suggest that the effect estimates for some of the recently discovered PD loci (i.e. *CCDC62*/*HIP1R*, *MCC1*, and *STK39*) [21] may be comparable in Caucasian and Asian populations (Table S1), although additional datasets are needed to establish genome-wide significance in populations of Asian-descent for these loci. Conversely, only insufficient data are currently available to assess the effect sizes of *GAK* and *SYT11/RAB25* on PD risk in Asians: *GAK* rs6599388 violated Hardy-Weinberg equilibrium in Asian datasets from the GEO-PD consortium and was thus excluded from further analyses on that ethnic group [23]. *SYT11/RAB25* chr1:154105678 was excluded from all analyses due to technical reasons in the study by the GEO-PD consortium [23]. Moreover, none of the reported *SYT11/RAB25* and *GAK* SNPs from the recent GWAS meta-analysis [21] were captured directly or by proxy (with an r^2^≥0.8) in the Japanese GWAS dataset [14], [23]. Finally, Asian-descent populations cannot be appropriately assessed for PD association with the *MAPT*-H1/H2 haplotype, rs10928513 in *ACMSD*, and rs7077361 in *ITGA8* owing to monomorphicity at these sites [14], [23].

### Evaluating the credibility of significant associations

To estimate the epidemiologic credibility of associations with polymorphisms showing sub-genome-wide significant association with PD (*P*>5×10^−8^), we applied two “credibility” measures for each such result. First, we calculated Bayes factors (BF, expressed here as log_10_-values, “logBF”) assuming an average non-null odds ratio of 1.15, as approximation of a typical “complex disease effect size”, and a spike and smear prior distribution of effects [25]. Our second assessment was based on the Human Genome Epidemiology Network's (HuGENet) interim criteria for the assessment of cumulative epidemiologic evidence in genetic association studies [26], [27]. The results of these analyses are summarized in Table S1.

There was strong epidemiologic support in both assessments for all loci showing genome-wide significant association. This included several additional polymorphisms in these same loci that only showed sub-genome-wide significant association. However, there was no additional sub-genome-wide significantly associated locus that received unequivocally strong support from both credibility assessments (Table S1). In this list, the strongest support was assigned to SNP chr6:32588205 in the *HLA* locus receiving the best possible grade in the HuGENet criteria (grade A), but more moderate support in the Bayesian analyses (logBF = 4.4). However, the relevance of this assessment needs to be evaluated as the underlying analysis was only based on four GWAS datasets.

## Discussion

The PDGene database represents a comprehensive, regularly updated and freely available online research synopsis of genetic association studies in PD. Detailed summaries of the most compelling findings are provided within an easy-to-use, dedicated online framework, displaying forest plots, cumulative meta-analyses, and an up-to-date ranking of “Top Results”. To allow comparison of PDGene results with association findings from other complex diseases and to facilitate their interpretation with respect to functional genetics data, all meta-analysis results have been ported as a customized track onto the UCSC Genome Browser. This will also allow for a integration and visualization [28] of association results from large-scale resequencing data (e.g. from whole-exome or whole-genome studies) into PDGene once these become available.

To the best of our knowledge, our study represents the most comprehensive research synopsis in the field of PD genetics. In addition, it represents the first disease-specific genetic database that allows a systematic and exhaustive inclusion of GWAS data, and may serve as a model for similar databases in other complex genetic diseases. Owing to our multi-pronged data retrieval and analysis protocol we were able to perform meta-analyses on the vast majority of PD risk-gene candidates, including those “featured” as top association results in all published GWAS. In particular, this includes the five novel loci recently featured in the recent GWAS meta-analysis [21]. Through collaboration with other PD genetics laboratories we obtained independent summary data for these and 142 additional SNPs, substantially extending the hitherto available evidence. Taken together, our analyses provide unequivocal evidence that *BST1*, *CCDC62/HIP1R*, *DGKQ/GAK*, *GBA*, *ITGA8*, *LRRK2*, *MAPT*, *MCCC1/LAMP3*, PARK16, *SNCA*, *STK39*, *SYT11/RAB25* represent genuine PD risk loci, while the role of several other loci (e.g. *ACMSD/TMEM163*, and the *HLA* locus) remains to be determined. The unpublished data aggregated here from various PD genetics groups for selected candidate genes represents the first step towards a systematic meta-analysis across the full GWAS datasets from the same populations. Once completed, the results of this “mega” meta-analysis will be posted on the PDGene database, allowing users to browse the complete results via the customized genome browser track already in place.

Of particular interest are loci with unusually large effect sizes. While most loci in PDGene have only small effects on PD risk (with ORs ranging from 1.10 to 1.35, which are typical for complex diseases), for some loci much larger ORs were estimated (i.e. *GBA* [OR 3.51 in Caucasians], *LRRK2* [OR 2.23 in Asians], and *SYT11*/*RAB25* [OR 1.73 in Caucasians], see Table 2). The risk-allele frequencies at these polymorphisms are typically rather small (i.e. below 0.05), resulting in low population attributable risks for these loci (for the above mentioned loci individually less than 2%).

Interestingly, the meta-analysis results of *GBA* N370S as well as the *LRRK2* rs34778348 are solely based on candidate-gene approaches since these SNPs are not on any of the current GWAS arrays or imputation reference panels. Thus, even in the “GWAS era” smaller-scale, non-GWAS but “focused” genetic studies, will likely continue to play an important role. This is also true when it comes to providing independent replication of proposed disease associations and/or when validating imputation-derived results by direct genotyping in sufficiently sized datasets. PDGene systematically concatenates all these different types of data into one database framework, vastly facilitating an assessment of the overall evidence for any given SNP or locus.

The strength of our approach is further exemplified by the identification of genome-wide significant association between disease risk and a SNP in *ITGA8*, which was not featured as a relevant PD gene in any previous study. *ITGA8* (encoding integrin alpha 8, a type-I transmembrane protein) is functionally interesting as it is expressed in brain [29], mediates cell-cell interactions and regulates neurite outgrowth of sensory and motor neurons [30]. Additional studies are needed to further assess the potential role of this gene in PD pathogenesis. Furthermore, PDGene shows that two additional loci, not highlighted by the recent GWAS meta-analysis [21], yield genome-wide signficiant results in the PDGene meta-analyses, i.e. PARK16, originally implicated as a PD susceptibility locus in an Asian GWAS [14] but not highlighted in the recent GWAS meta-analysis on Caucasian samples [21] and *GBA*, a gene that was found soley by candidate-gene approaches. Another strength of our study is that it combines genetic data from currently more than 50 different countries allowing a systematic assessment of genetic associations across populations of different ethnic descent. For instance, these analyses suggest that variants in *BST1*, *LRRK2*, the PARK16 locus, and *SNCA* show genome-wide significant association with PD risk in both Caucasian and Asian-descent samples. Furthermore, the recently described Caucasian GWAS loci *CCDC62*/*HIP1R*, *MCC1*, and *STK39* [21] also show similar effect size estimates in populations of Asian-descent [23]. PD association data originating from other ethnic groups are still relatively scarce. However, they could easily be added to the already existing data on the respective polymorphisms available on PDGene.

In summary, we have created a continuously updated online resource for genetic association studies in the field of PD. Synthesizing essentially all available data in the field led to the identification of *ITGA8* as a novel potential PD risk locus. Our quantitative approach to data integration across a multitude of different study designs can be readily scaled to include large-scale resequencing efforts that will emerge over the coming years, making the complex field of PD genetics accessible to a broad range of investigators.

## Methods

Note that the following section only provides a brief summary of the methods applied to our study. A much more detailed description can be found in Text S1.

### Literature searches

#### Inclusion criteria

For inclusion in PDGene, a study has to meet three criteria: 1) It must evaluate the association between a bi-allelic genetic polymorphism (minor allele frequency ≥0.01 in the healthy control population of at least one study) and Parkinson's disease (PD) risk in datasets comprised of both affected (defined as clinically and/or neuropathologically diagnosed “Parkinson's disease”) and unaffected individuals; 2) it must be published in a peer-reviewed journal; 3) it must be published in English. For this manuscript, we also included data on ten SNPs generated in the GEO-PD Consortium datasets [14], [23] and obtained data for the newly identified SNP rs7077361 in *ITGA8* from the Japanese GWAS dataset [14].

#### Exclusion criteria

In brief, genetic association data of the following studies were excluded from the meta-analyses (see Text S1 for details): family-based studies without available subject-level data (however, unrelated case-control data enriched for familial cases were not excluded), studies investigating only disease controls, multi-allelic polymorphisms, and studies of polymorphisms in mitochondrial DNA. We also excluded genetic data of apparently “poor” quality if discrepancies could not be resolved after contacting the study authors (e.g. inadequate genotyping/sequencing protocols or discrepancies in terms of allele names or frequencies when compared with public databases; more details can be found in Text S1).

#### Search strategies

**Figure 3:**
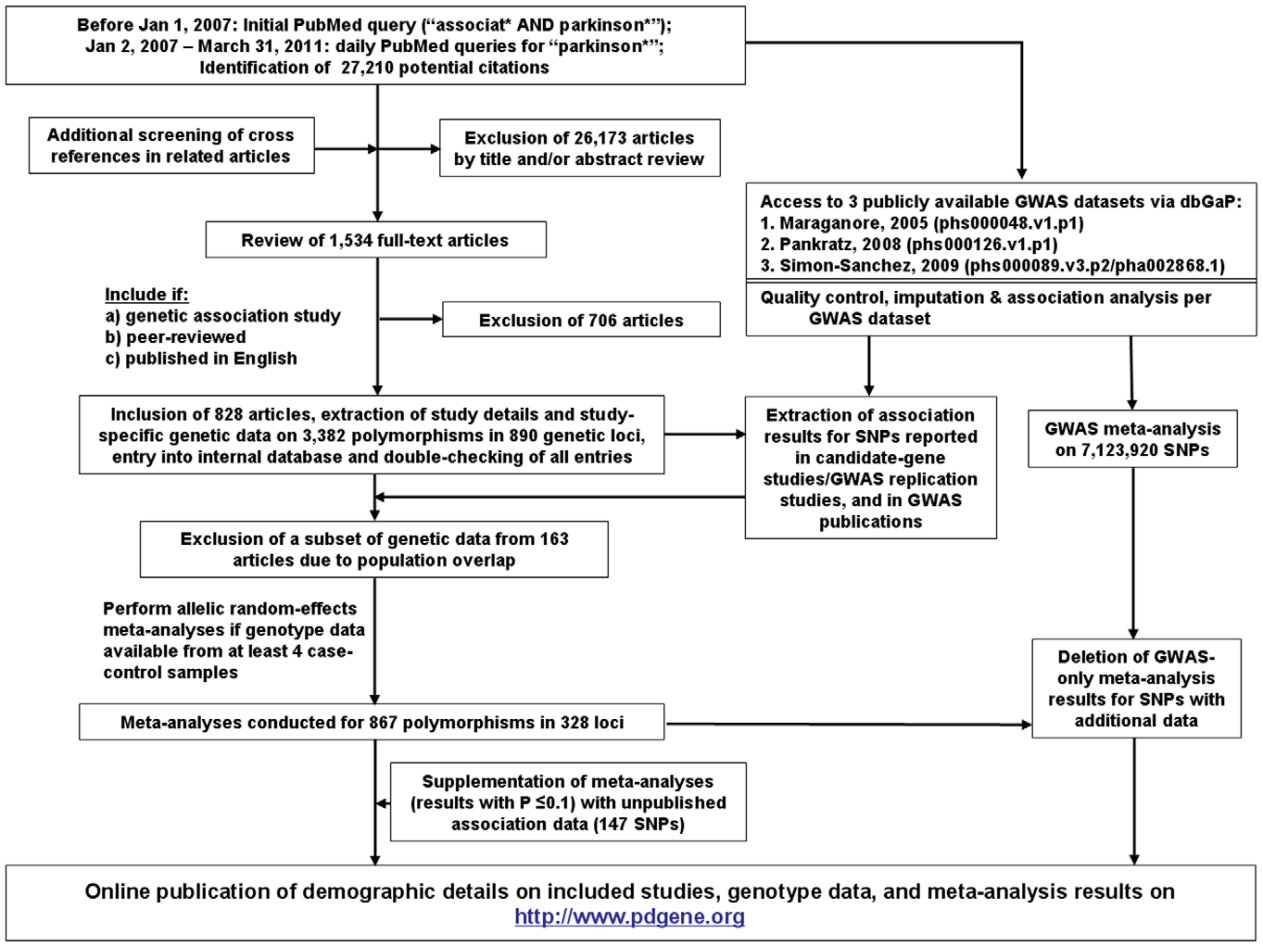
Flowchart of literature search, data extraction, and analysis strategies applied for PDGene.

Our literature searches until March 31^st^, 2011, yielded 27,210 articles, which were screened for eligibility using the title, abstract, or full-papers, as necessary. Additional screening of bibliographies in reviews, published meta-analyses, and original genetic association studies were also performed. Overall, full text versions of 1,534 articles were obtained. Following the inclusion and exclusion criteria outlined above, 828 articles were included in PDGene until March 31^st^ 2011 (also see Figure 3).

### Statistical analyses

#### Meta-analyses

Random-effects allelic meta-analyses [31] were performed if a minimum of four independent datasets existed per polymorphism. Summary odds ratios [ORs] and 95% confidence intervals [CIs] were calculated irrespective of ethnic descent as well as for distinct ethnic groups (i.e. Caucasians, and Asians) if sufficient data were available. In addition, we performed a number of sensitivity analyses (excluding the initial studies and datasets in which HWE was violated in control individuals), systematically assessed between-study heterogeneity (via *I* ^2^), and assessed the credibility of each at least nominally significant meta-analysis result by calculating Bayes factors (BF; here expressed as log10(BF)="logBF”) [25] and by determining a grading score developed by the Human Genome Epidemiology Network (HuGENet) [26], [27].

#### Assessment of small-study bias/publication bias

This is of particular importance in meta-analyses of published association data and was carefully addressed here: First, we added *publicly* available GWAS data [10], [12], [13] to the vast majority of SNPs. Since these data are typically unbiased, this should decrease the potential for small-study bias/publication bias. Secondly, for 147 SNPs of the core PDGene meta-analyses that showed statistically suggestive results (*P*≤0.1), we obtained additional data from all currently published, but *not publicly* available GWAS datasets, further decreasing a potential impact of small-study bias/publication bias. Thirdly, we directly assessed the evidence for small study bias by applying a recently proposed regression test [32] on all nominally significant (*P*<0.05) meta-analysis results. The results of these analyses are fully displayed in Table S1.

#### GWAS-only meta-analyses

We obtained individual-level genotype data for all publicly available PD GWAS datasets from NCBI's “dbGAP” database (a total of three [10], [12], [13] at the time of the datafreeze, March 31^st^, 2011). Genotype data were cleaned using standard procedures, followed by imputation of untested genotypes (using reference panels from HapMap and the 1000 Genomes Project), and association analyses incorporating imputation uncertainty (case-control datasets only), age, sex, and population stratification. Overall, this procedure led to a total of 7,723,931 unique SNPs, 7,123,920 of which were present in at least two, and 711,271 in at least three datasets. Meta-analyses (either combining test-statistics and standard errors using random-effects models, or by combining P-values weighted by sample size, see Text S1 for more details) were performed on the 7,123,920 SNPs present in at least two of the GWAS datasets.

### Online database

After completion of all data-management and analysis steps, all study-specific variables, genotype data (except for GWAS), and meta-analysis plots are posted on a dedicated, publicly available, online adaptation of the PDGene database using the same software and code as our databases for Alzheimer's disease [33] and schizophrenia [34]. The online database is hosted by the “Alzheimer Research Forum” and can be accessed via its own designated URL (http://www.pdgene.org).

### Database code

The database software can easily be ported to other genetically complex diseases and will be made available on a collaborative basis to interested researchers upon request.

## Supporting Information

Figure S1 QQ plots showing the distribution of expected versus observed P-values for the GWAS-only meta-analysis results. Analyses were performed using the METAL software (ref. [21] in Text S1). The excess of observed *P*-values (Figure S1, panel 1) is entirely due to association signals in the *SNCA*, *MAPT*, *LRRK2*, and *DGKQ*/*GAK* loci as can be seen in Figure S1, panel 2 that showcases the *P*-value distributions after removal of 18,622 SNPs in these regions (lambda = 1.007).
(TIF)

Figure S2 Forest plots of allelic meta-analyses for SNPs showing genome-wide significant association (P<5×10−8) with PD susceptibility in the March 31^st^ 2011 datafreeze. Study-specific allelic odds ratios (ORs, black squares) and 95% confidence intervals (CIs, lines) were calculated for each included dataset. The summary OR and CI was calculated using random-effects models (grey diamond). Whenever multiple polymorphisms showed genome-wide significant association in the same locus, only the variant with the smallest P-value is listed here for meta-analysis results after stratification for Caucasian and Asian ancestries. For a complete list of meta-analyses performed for the datafreeze, see Table S1. Figure S1, panel 1-S1, panel 12 and S1, panel 13-S1, panel 16 display the SNP showing the most significant genome-wide association in datasets of Caucasian ancestry and Asian ancestry, respectively. Details and references of all included studies displayed here can be found on the PDGene database (http://www.pdgene.org). I2 = estimate of percentage of between-study heterogeneity that is beyond chance, “excl initial” = summary OR and 95%CI after meta-analysis after exclusion of the initial study, C = Caucasian ancestry, A = Asian ancestry, H = Hispanic descent, D = African descent, “•” = initial study (applies to candidate-gene studies), “†” = no data provided or data was not eligible for inclusion in meta-analysis, “‡” = study excluded due to overlap, “#” = HWE violation in controls (P<0.05, not applicable to quality-controlled GWAS datasets, see Text S1), “i” = SNP monomorphic in the respective dataset, “ø” = meta-analysis after excluding initial study not applicable.
(PDF)

Figure S3 Locus plot of the ITGA8 region on chromosome 10p13 (15346353–15801533 bp, hg18). The figure displays association results for ∼1,400 SNPs in the *ITGA8* region including at least four independent datasets. SNPs are color-coded based on linkage disequilibrium (r^2^) estimates from the CEU 1000G dataset (release June 2010). All LD estimates refer to the most significantly associated SNP rs7077361. SNPs color-coded in grey indicate missing LD estimates in the CEU dataset. Recombination rates were estimated based on the CEU dataset, and are displayed as blue line in the background. Gene annotations are based on RefSeq and the UCSC Genome browser. Locus plots were generated using the LocusZoom Stand-alone package (http://genome.sph.umich.edu/wiki/LocusZoom_Standalone).
(TIF)

Figure S4 Forest plots of fixed-effect meta-analyses for SNP rs6723108 in the ACMSD/TMEM163 locus and chr6:32609909 in the HLA locus. Symbols are the same as for Figure S2 (see above).
(TIF)

Table S1 Overview of all 867 polymorphisms meta-analyzed in the March 31^st^ 2011 datafreeze using random-effects allelic models. Random-effects allelic meta-analyses were performed on polymorphisms for which four or more independent datasets were available. Meta-analyses after stratification for different ethnic descent were performed if at least three independent datasets were available in the respective stratum (applicable only to samples of European and Asian descent). Each nominally significant meta-analysis result (*P*<0.05) was graded according to the HuGENet interim criteria. For details on how these criteria are applied, see Text S1. Meta-analysis results in this table are ordered by genomic location. OR = Odds Ratio, CI = confidence interval, N minor = number of minor alleles, Ethnicities: C = Caucasian, A = Asian, D = African Descent, H = Hispanic, O = Other/Mixed, Low OR = OR<1.15 or ≥0.87, respectively, F = loss of significance in the respective meta-analysis after exclusion of the first study, HWE = loss of significance after excluding studies violating HWE (*P*<0.05), Regr = evidence for small-study/publication bias using a modified regression test (see Text S1), A = Grade A (‘strong’ epidemiologic credibility), B = Grade B (‘modest’ epidemiologic credibility), C = Grade C (‘weak’ epidemiologic credibility), logBF = Bayes Factor (see Text S1). “*” denotes SNPs that have been supplemented by additional data after the datafreeze (in total this applies to 147 SNPs, see Text S1 for the description of included datasets).
(XLS)

Table S2 Investigation of the extent of statistical inflation assuming sample overlaps of 1%, 5%, and 10% across cases and controls in datasets originating from the same countries. Hypothetical sample overlap across datasets was assumed between different candidate-gene/replication studies and between candidate-gene/replication studies and GWAS datasets if they originated from the same country. These analyses were performed applying random-effects models and adding the sum of weighted co-variances of overlapping datasets to the overall study variance (see ref. [24] in the main text). Note that the assumption of undetected overlapping samples does not apply (and was therefore not modeled here) to overlap between individual GWAS as duplicate samples in these datasets were removed prior to meta-analysis. It also does not apply to independent datasets used in the same publication where duplicate samples had been removed by the authors prior to analysis and publication. We emphasize that this table describes hypothetical scenarios, because the geographical origin of each study had been investigated extensively and potentially overlapping datasets had been excluded as part of PDGene's data inclusion protocol. Thus, the extent of overlap across geographically distinct datasets within the same countries is reduced to accidental recruitment of the same subjects more than once in different datasets throughout the respective countries, and can be expected to be less than ∼1%. This estimate is based on data of the GEO-PD consortium, where sufficient data were centrally available of 6,072 subjects from 20 geographically distinct sites in 13 countries that had been investigated for potentially duplicate samples across sites, but no duplicate subjects (neither between not within countries) were identified when matching on ethnicity, birth, sex, and genotype. The investigation of overlap was not applicable here for Asian datasets, as they originated from different countries and/or were cleaned by the respective authors prior to publication.
(DOC)

Text S1 Supplementary material. This file includes supplementary methods and references as well as the list of members of the GWAS consortia, the GEO-PD Consortium, and consortia-specific acknowledgements.
(PDF)
