# Supplementary figures and images for: Comprehensive Research Synopsis and Systematic Meta-Analyses in Parkinson's Disease Genetics: The PDGene Database
Source: PLoS Genet. 2012 Mar 15;8(3):e1002548. doi: 10.1371/journal.pgen.1002548 (PMC3305333; doi:10.1371/journal.pgen.1002548)

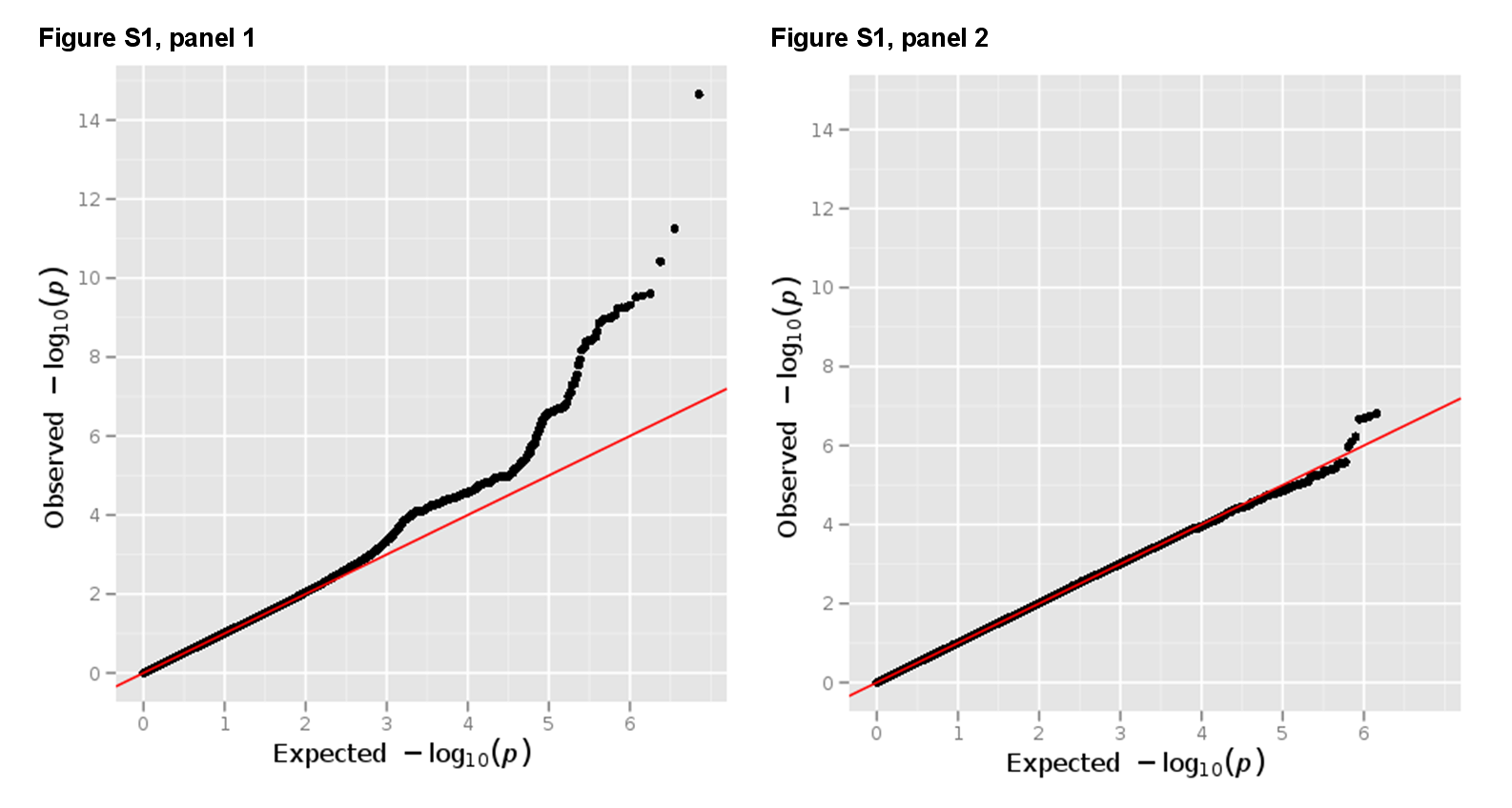

Supplement: Figure S1 — QQ plots showing the distribution of expected versus observed P-values for the GWAS-only meta-analysis results. Analyses were performed using the METAL software (ref. [21] in Text S1). The excess of observed P-values (Figure S1, panel 1) is entirely due to association signals in the SNCA, MAPT, LRRK2, and DGKQ/GAK loci as can be seen in Figure S1, panel 2 that showcases the P-value distributions after removal of 18,622 SNPs in these regions (lambda = 1.007). (TIF) [file pgen.1002548.s001.tif]

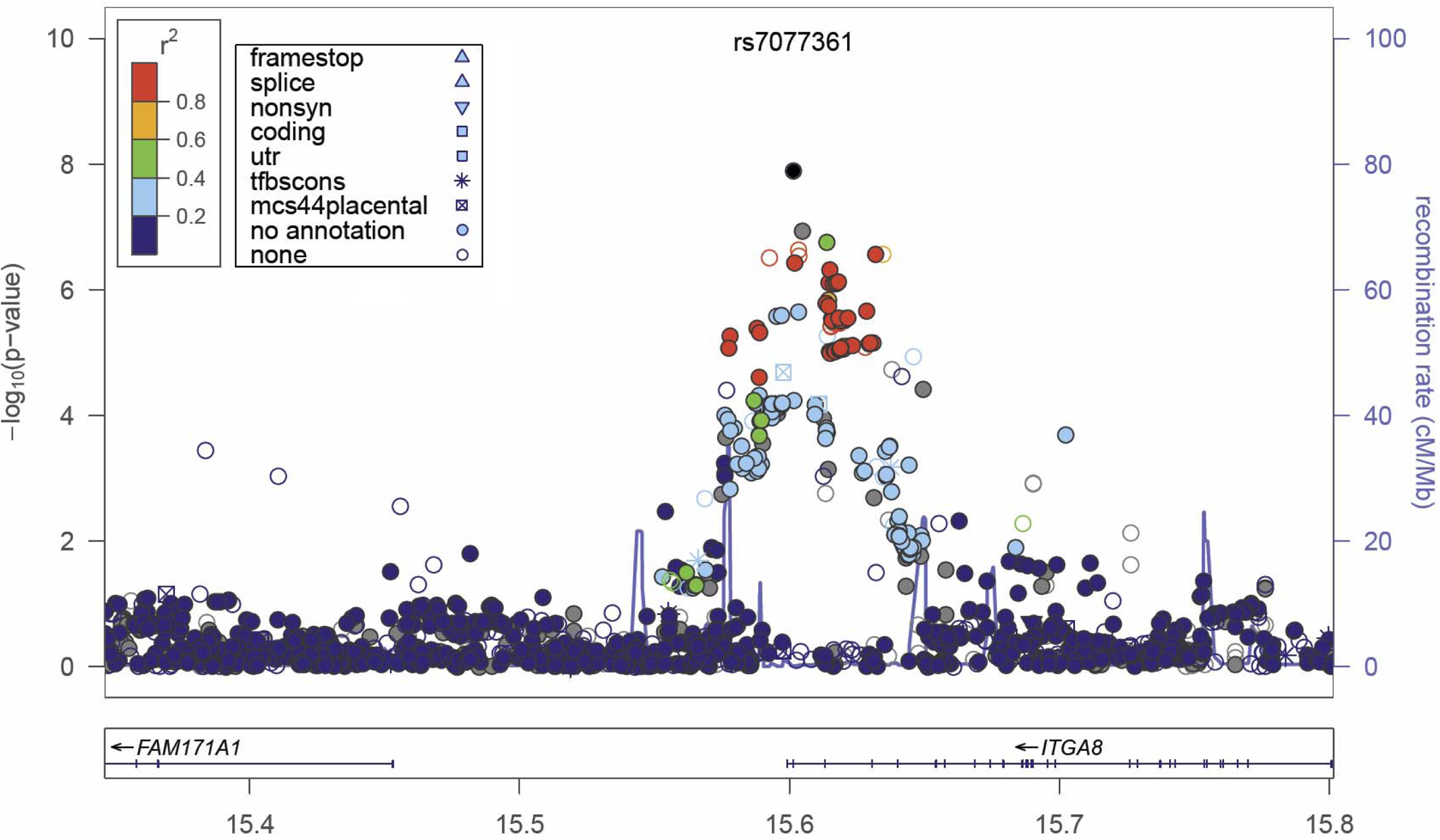

Supplement: Figure S3 — Locus plot of the ITGA8 region on chromosome 10p13 (15346353–15801533 bp, hg18). The figure displays association results for ∼1,400 SNPs in the ITGA8 region including at least four independent datasets. SNPs are color-coded based on linkage disequilibrium (r2) estimates from the CEU 1000G dataset (release June 2010). All LD estimates refer to the most significantly associated SNP rs7077361. SNPs color-coded in grey indicate missing LD estimates in the CEU dataset. Recombination rates were estimated based on the CEU dataset, and are displayed as blue line in the background. Gene annotations are based on RefSeq and the UCSC Genome browser. Locus plots were generated using the LocusZoom Stand-alone package (http://genome.sph.umich.edu/wiki/LocusZoom_Standalone). (TIF) [file pgen.1002548.s003.tif]

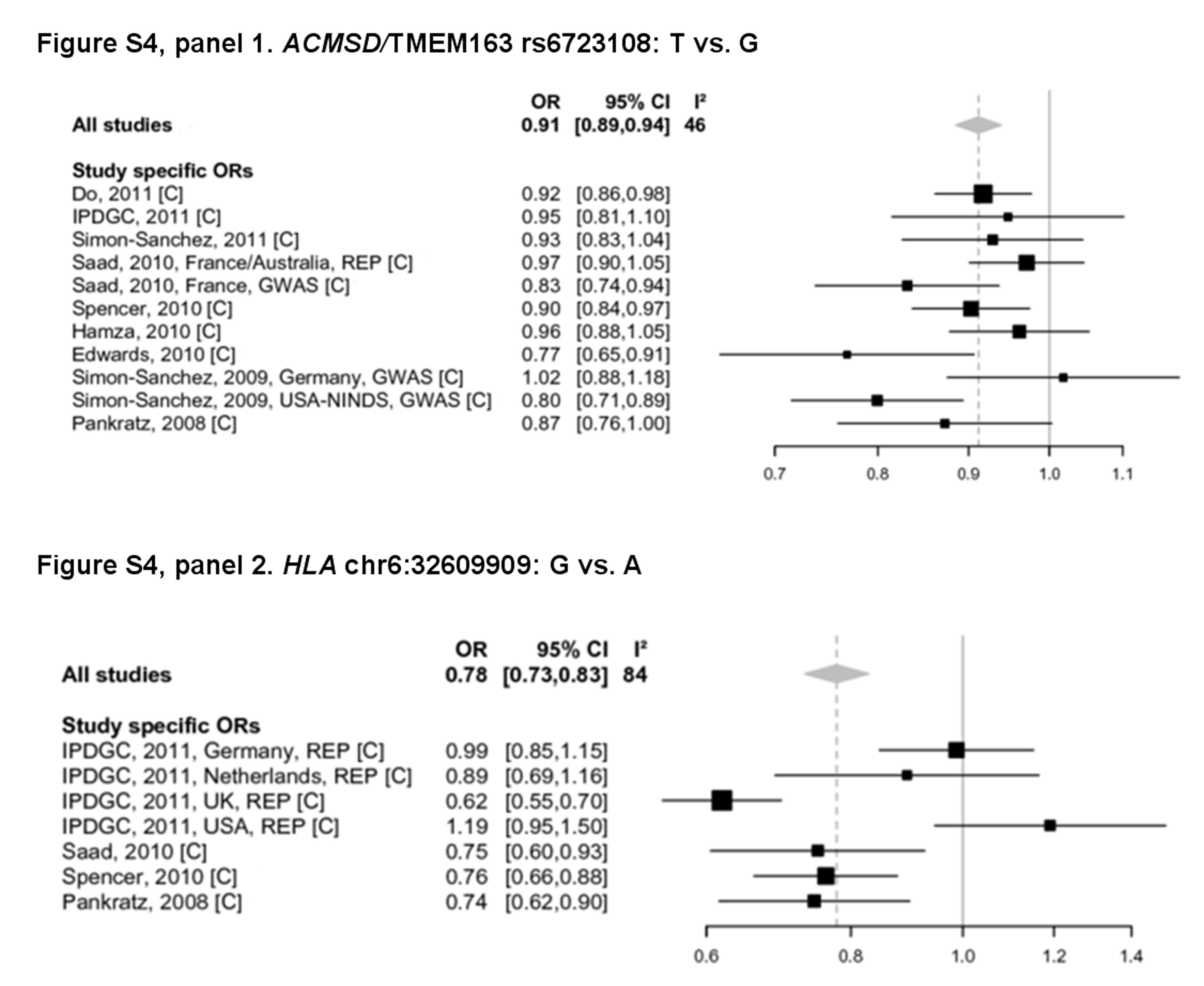

Supplement: Figure S4 — Forest plots of fixed-effect meta-analyses for SNP rs6723108 in the ACMSD/TMEM163 locus and chr6:32609909 in the HLA locus. Symbols are the same as for Figure S2 (see above). (TIF) [file pgen.1002548.s004.tif]
