# Supplementary material for: Comprehensive Research Synopsis and Systematic Meta-Analyses in Parkinson's Disease Genetics: The PDGene Database
Source: PLoS Genet. 2012 Mar 15;8(3):e1002548. doi: 10.1371/journal.pgen.1002548 (PMC3305333; doi:10.1371/journal.pgen.1002548)

Figure S2, panel 1. *GBA* N370S: G vs. A

	OR	95% CI	I²
**All studies**	**3.45**	**[2.70, 4.40]**	**15**
**All excl initial**	**3.42**	**[2.67, 4.38]**	**17**
**Caucasian studies**	**3.51**	**[2.55, 4.83]**	**38**
**Caucasian excl initial**	**3.46**	**[2.50, 4.79]**	**40**
**Study specific ORs**			
Do, 2011 [C]	4.05	[3.08, 5.32]	
Lesage, 2011 [O]	3.67	[0.17, 81.74]	
Lesage, 2010 [C]	6.49	[1.56, 27.00]	
Santos, 2010 [O]	7.76	[0.39,155.65]	
Hu, 2010 [A]	2.76	[0.55, 13.73]	
Sun, 2010 [A] ¡	–	–	
Nishioka, 2009 [O]	0.52	[0.05, 4.97]	
Sidransky, 2009, Japan [A] ¡	–	–	
Sidransky, 2009, Singapore [A] ¡	–	–	
Sidransky, 2009, Taiwan [A] ¡	–	–	
Sidransky, 2009, France [C]	6.84	[0.85, 54.87]	
Sidransky, 2009, Germany (Rostock) [C]	3.58	[0.42, 30.74]	
Sidransky, 2009, Germany (Tuebingen) [C]	12.19	[0.69,215.25]	
Sidransky, 2009, Italy [C] ‡	–	–	
Sidransky, 2009, Canada [C] ‡	–	–	
Sidransky, 2009, Norway [C] ‡	–	–	
Sidransky, 2009, Portugal [C]	3.50	[0.83, 14.73]	
Sidransky, 2009, Brazil [O]	8.19	[0.27,245.59]	
Sidransky, 2009, USA [O] ‡	–	–	
Sidransky, 2009, Israel [O] ‡	–	–	
Sidransky, 2009, USA (NHGRI) [O]	3.53	[0.82, 15.29]	
Sidransky, 2009, USA (Seattle) [O]	4.50	[1.02, 19.85]	
Neumann, 2009 [C]	2.61	[0.33, 20.92]	
Nichols, 2008 [C]	1.60	[0.40, 6.42]	
Gan-Or, 2008 [C]	2.31	[1.70, 3.13]	
Mata, 2008 [C] ‡	–	–	
De Marco, 2008 [C]	7.37	[0.37,147.32]	
Bras, 2007 [C] ‡	–	–	
Clark, 2007 [C]	5.57	[1.67, 18.59]	
Toft, 2006 [C]	0.87	[0.25, 2.98]	
Eblan, 2006 [O]	1.80	[0.06, 54.65]	
Sato, 2005 [C]	1.39	[0.09, 22.35]	
Clark, 2005 [C] ‡	–	–	
Aharon-Peretz, 2004 [C]	5.58	[3.58, 8.72]	
Lwin, 2004 [C] •	11.80	[0.66,210.85]	

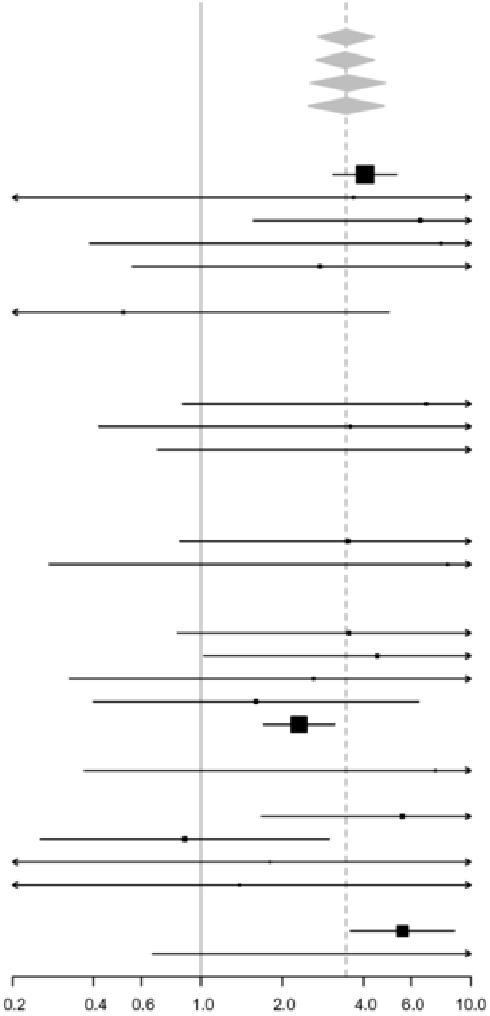

Figure S2, panel 2. *SYT11/RAB25* chr1:154105678: T vs. C

	OR	95% CI	I²
**All studies**	**1.73**	**[1.48,2.01]**	**0**
**Study specific ORs**			
Simon-Sanchez, 2011 [C]	1.97	[1.41,2.75]	
Saad, 2010 [C]	1.96	[1.20,3.19]	
Spencer, 2010 [C]	1.50	[1.16,1.94]	
Simon-Sanchez, 2009, Germany, GWAS [C]	1.97	[1.21,3.20]	
Simon-Sanchez, 2009, USA-NINDS, GWAS [C]	1.57	[1.04,2.37]	
Pankratz, 2008 [C]	1.91	[1.17,3.12]	

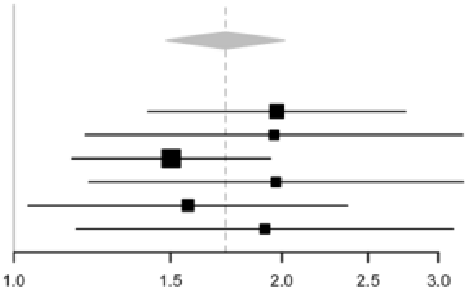

Figure S2, panel 3. PARK16 rs947211: A vs. G

	OR	95% CI	I²
**All studies**	**0.88**	**[0.85,0.91]**	**39**
**Caucasian studies**	**0.91**	**[0.88,0.94]**	**0**
**Asian studies**	**0.79**	**[0.73,0.85]**	**29**
**Study specific ORs**			
Mata, 2011 [C]	0.95	[0.83,1.09]	
Do, 2011 [C]	0.87	[0.83,0.93]	
IPDGC, 2011 [C]	0.91	[0.77,1.08]	
Simon-Sanchez, 2011 [C]	0.89	[0.77,1.02]	
Saad, 2010 [C]	0.85	[0.75,0.97]	
Spencer, 2010 [C]	0.95	[0.86,1.04]	
Ramirez, 2010 [H]	0.84	[0.63,1.13]	
Hamza, 2010 [C]	0.90	[0.80,1.00]	
Tan, 2010 [A]	0.88	[0.75,1.04]	
Edwards, 2010 [C]	0.94	[0.77,1.14]	
Satake, 2009, Japan, Replication-1 [A]	0.74	[0.66,0.83]	
Satake, 2009, Japan, Replication-2 [A]	0.73	[0.62,0.87]	
Satake, 2009, Japan, GWAS [A]	0.82	[0.74,0.90]	
Simon-Sanchez, 2009, USA/Germany/UK, Stage II [C]	0.96	[0.88,1.04]	
Simon-Sanchez, 2009, Germany, GWAS [C]	0.84	[0.71,1.00]	
Simon-Sanchez, 2009, USA-NINDS, GWAS [C]	0.90	[0.80,1.02]	
Pankratz, 2008 [C]	0.97	[0.83,1.14]	
Fung, 2006 [C] ‡	–	–	

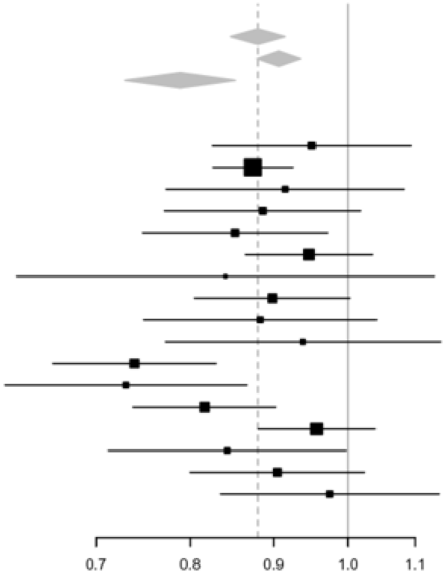

Figure S2, panel 4. *STK39* rs2390669: C vs. A

	OR	95% CI	I²
**All studies**	**1.19**	**[1.12,1.25]**	**18**
**Study specific ORs**			
IPDGC, 2011, France, REP [C]	1.07	[0.77,1.49]	
IPDGC, 2011, Germany, REP [C]	1.11	[0.91,1.36]	
IPDGC, 2011, Netherlands, REP [C]	1.35	[0.99,1.82]	
IPDGC, 2011, UK, REP [C]	1.18	[1.02,1.36]	
IPDGC, 2011, USA, REP [C]	1.12	[1.00,1.25]	
IPDGC, 2011, Iceland, GWAS [C]	0.79	[0.59,1.06]	
Simon-Sanchez, 2011 [C]	1.11	[0.90,1.35]	
Saad, 2010 [C]	1.40	[1.17,1.68]	
Spencer, 2010 [C]	1.22	[1.07,1.39]	
Hamza, 2010 [C]	1.19	[1.04,1.36]	
Edwards, 2010 [C]	1.22	[0.93,1.59]	
Simon-Sanchez, 2009, Germany, GWAS [C]	1.36	[1.05,1.76]	
Simon-Sanchez, 2009, USA-NINDS, GWAS [C]	1.19	[0.99,1.42]	
Pankratz, 2008 [C]	1.35	[1.07,1.71]	

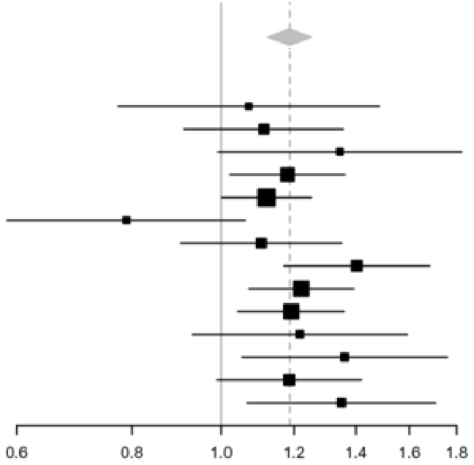

Figure S2, panel 5. *MCCC1/LAMP3* rs11711441: A vs. G

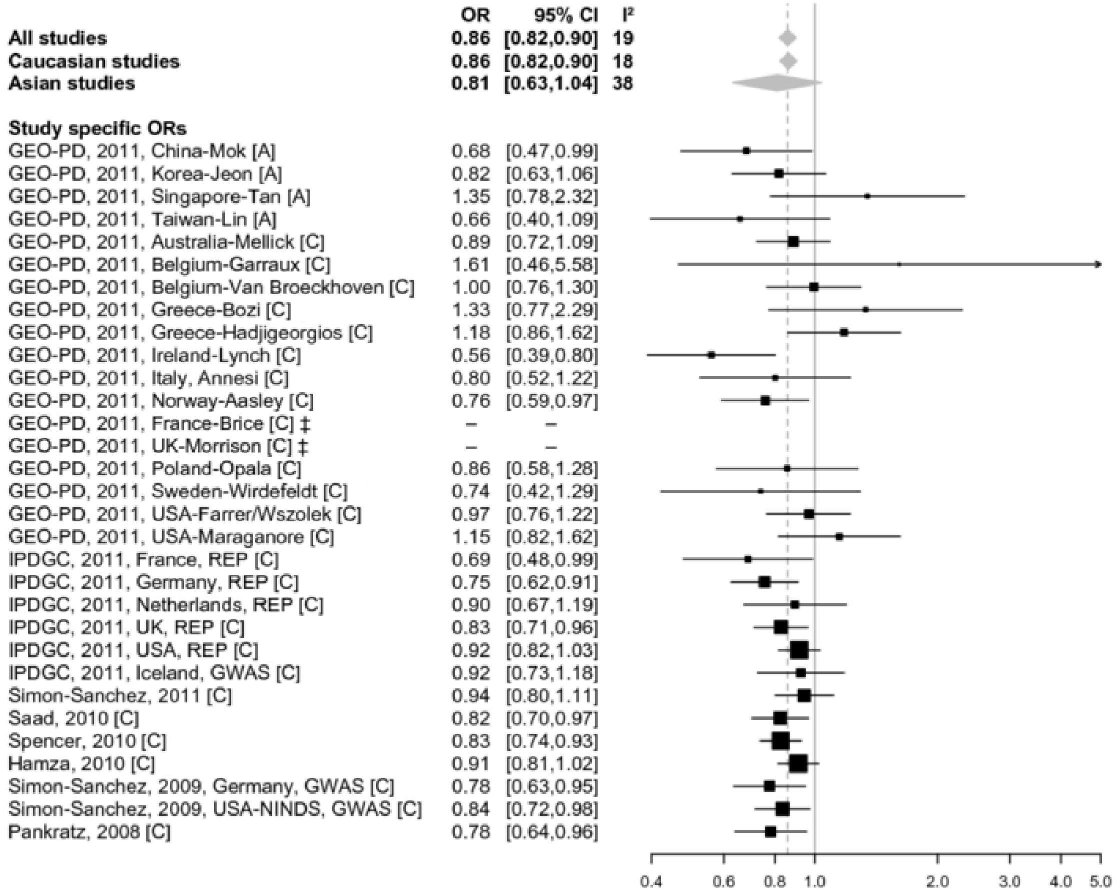

Figure S2, panel 6. *DGKQ/GAK* rs11248060: T vs. C

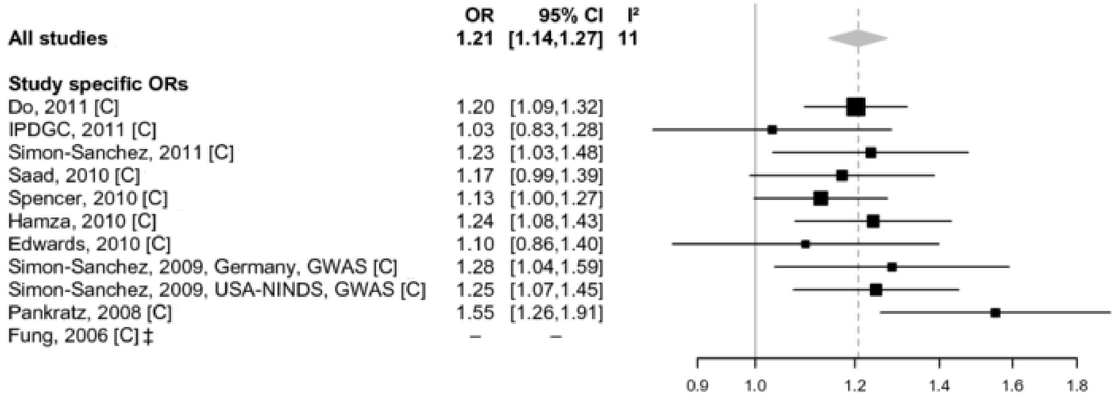

Figure S2, panel 7. *BST1* rs11724635: C vs. A

	OR	95% CI	I^2
**All studies**	**0.87**	**[0.84,0.91]**	**46**
**Caucasian studies**	**0.88**	**[0.84,0.91]**	**43**
**Asian studies**	**0.79**	**[0.61,1.04]**	**55**
**Study specific ORs**			
GEO-PD, 2011, China-Mok [A]	0.88	[0.66,1.18]	
GEO-PD, 2011, Korea-Jeon [A]	0.65	[0.52,0.80]	
GEO-PD, 2011, Taiwan-Lin [A]	1.01	[0.63,1.62]	
GEO-PD, 2011, Australia-Mellick [C]	0.98	[0.86,1.12]	
GEO-PD, 2011, Belgium-Garraux [C]	1.19	[0.56,2.53]	
GEO-PD, 2011, Belgium-Van Broeckhoven [C]	0.85	[0.70,1.03]	
GEO-PD, 2011, Greece-Bozi [C]	0.52	[0.34,0.79]	
GEO-PD, 2011, Greece-Hadjigeorgios [C]	1.19	[0.95,1.49]	
GEO-PD, 2011, Ireland-Lynch [C]	0.84	[0.67,1.04]	
GEO-PD, 2011, Italy, Annesi [C]	0.82	[0.61,1.11]	
GEO-PD, 2011, Norway-Aasley [C]	0.86	[0.72,1.03]	
GEO-PD, 2011, France-Brice [C] ‡	–	–	
GEO-PD, 2011, UK-Morrison [C] ‡	–	–	
GEO-PD, 2011, Poland-Opala [C]	0.95	[0.74,1.23]	
GEO-PD, 2011, Sweden-Wirdefeldt [C]	1.18	[0.83,1.67]	
GEO-PD, 2011, USA-Farrer/Wszolek [C]	0.87	[0.74,1.03]	
GEO-PD, 2011, USA-Maraganore [C]	0.87	[0.68,1.10]	
IPDGC, 2011, France, REP [C]	0.82	[0.65,1.03]	
IPDGC, 2011, Germany, REP [C]	0.84	[0.73,0.96]	
IPDGC, 2011, Netherlands, REP [C]	1.00	[0.81,1.23]	
IPDGC, 2011, UK, REP [C]	0.78	[0.71,0.87]	
IPDGC, 2011, USA, REP [C]	0.90	[0.83,0.97]	
IPDGC, 2011, Iceland, GWAS [C]	0.95	[0.80,1.13]	
Simon-Sanchez, 2011 [C]	0.88	[0.78,0.99]	
Saad, 2010 [C]	0.76	[0.69,0.85]	
Spencer, 2010 [C]	0.92	[0.85,1.00]	
Hamza, 2010 [C]	0.87	[0.80,0.96]	
Edwards, 2010 [C]	0.87	[0.73,1.03]	
Simon-Sanchez, 2009, Germany, GWAS [C]	0.84	[0.73,0.97]	
Simon-Sanchez, 2009, USA-NINDS, GWAS [C]	0.97	[0.87,1.07]	
Pankratz, 2008 [C]	0.75	[0.64,0.87]	

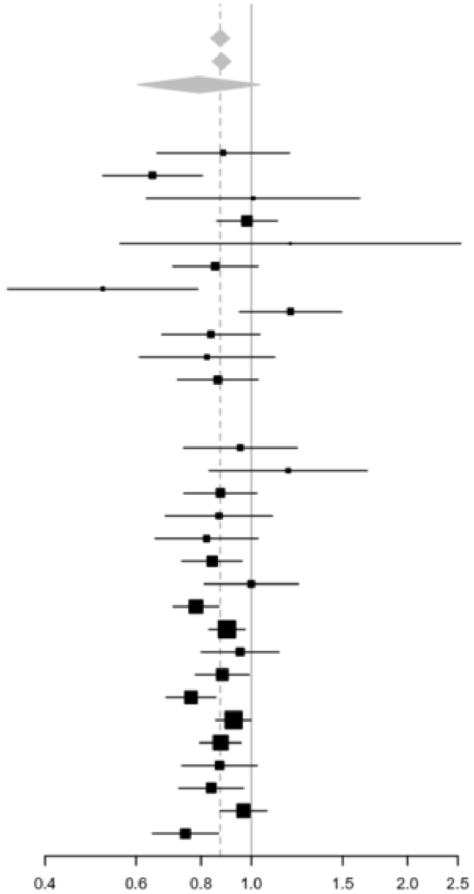

Figure S2, panel 8. *SNCA* rs356219: G vs. A

	OR	95% CI	I²
**All studies**	**1.30**	**[1.26,1.33]**	**18**
**Caucasian studies**	**1.29**	**[1.25,1.33]**	**16**
**Asian studies**	**1.33**	**[1.11,1.59]**	**34**
**Study specific ORs**			
GEO-PD, 2011, China-Mok [A]	1.16	[0.87,1.55]	
GEO-PD, 2011, Korea-Jeon [A] ‡	–	–	
GEO-PD, 2011, Singapore-Tan [A]	1.45	[0.94,2.24]	
GEO-PD, 2011, Taiwan-Lin [A]	1.03	[0.68,1.56]	
GEO-PD, 2011, Australia-Mellick [C]	1.25	[1.09,1.44]	
GEO-PD, 2011, Belgium-Garraux [C]	1.27	[0.55,2.94]	
GEO-PD, 2011, Belgium-Van Broeckhoven [C]	1.08	[0.89,1.31]	
GEO-PD, 2011, Greece-Hadjigeorgios [C]	1.11	[0.88,1.39]	
GEO-PD, 2011, Ireland-Lynch [C]	1.80	[1.44,2.26]	
GEO-PD, 2011, Italy, Annesi [C]	1.31	[0.95,1.80]	
GEO-PD, 2011, Norway-Aasley [C]	1.18	[0.99,1.40]	
GEO-PD, 2011, Greece-Bozi [C] ‡	–	–	
GEO-PD, 2011, France-Brice [C] ‡	–	–	
GEO-PD, 2011, UK-Morrison [C] ‡	–	–	
GEO-PD, 2011, Poland-Opala [C]	1.29	[1.00,1.67]	
GEO-PD, 2011, Sweden-Wirdefeldt [C]	1.00	[0.69,1.45]	
GEO-PD, 2011, USA-Farrer/Wszolek [C]	1.27	[1.08,1.49]	
GEO-PD, 2011, USA-Maraganore [C]	1.25	[0.97,1.61]	
Mata, 2011 [C]	1.23	[1.09,1.38]	
Do, 2011 [C]	1.30	[1.21,1.40]	
IPDGC, 2011, France, REP [C]	1.44	[1.15,1.81]	
IPDGC, 2011, Germany, REP [C]	1.09	[0.95,1.25]	
IPDGC, 2011, Netherlands, REP [C]	1.32	[1.06,1.64]	
IPDGC, 2011, UK, REP [C]	1.29	[1.16,1.43]	
IPDGC, 2011, USA, REP [C]	1.28	[1.18,1.39]	
IPDGC, 2011, Iceland, GWAS [C]	1.29	[1.07,1.54]	
Simon-Sanchez, 2011 [C]	1.34	[1.19,1.51]	
Kim, 2010 [A]	1.50	[1.29,1.75]	
Elbaz, 2010, Greece-Bozi [C]	1.23	[0.84,1.81]	
Elbaz, 2010, Italy, Ferrarese [C]	1.67	[1.12,2.50]	
Elbaz, 2010, Italy, Valente [C]	1.50	[1.04,2.17]	
Elbaz, 2010, Australia-Mellick [C] ‡	–	–	
Elbaz, 2010, Greece-Hadjigeorgiou [C] ‡	–	–	
Elbaz, 2010, Ireland-Lynch [C] ‡	–	–	
Elbaz, 2010, Italy-Annesi [C] ‡	–	–	
Elbaz, 2010, Norway-Aasly [C] ‡	–	–	
Elbaz, 2010, Poland-Opala [C] ‡	–	–	
Elbaz, 2010, Sweden-Wirdefeldt [C] ‡	–	–	
Elbaz, 2010, USA-Wzsolek [C] ‡	–	–	
Elbaz, 2010, France-Chartier-Harlin [C] ‡	–	–	
Elbaz, 2010, Germany-Auberger [C] ‡	–	–	
Elbaz, 2010, Germany-Klein [C] ‡	–	–	
Elbaz, 2010, Germany-Krueger [C] ‡	–	–	
Wider, 2010, Ireland [C] ‡	–	–	
Wider, 2010, Norway [C] ‡	–	–	
Wider, 2010, USA [C] ‡	–	–	
Saad, 2010 [C]	1.35	[1.21,1.51]	
Mata, 2010, USA [C] ‡	–	–	
Mata, 2010, USA [C] ‡	–	–	
Botta-Orfila, 2010 [C] ‡	–	–	
Spencer, 2010 [C]	1.27	[1.17,1.37]	
Hamza, 2010 [C]	1.40	[1.27,1.54]	
Edwards, 2010 [C]	1.49	[1.25,1.77]	
Simon-Sanchez, 2009, Germany, GWAS [C] ¶	1.35	[1.17,1.56]	
Simon-Sanchez, 2009, USA-NINDS, GWAS [C] ¶	1.24	[1.12,1.38]	
Pankratz, 2009 [C] ‡	–	–	
Pankratz, 2008 [C]	1.41	[1.22,1.62]	
Westerlund, 2008 [C]	1.17	[0.91,1.52]	
Myhre, 2008 [C] ‡	–	–	
Goris, 2007 [C] ‡	–	–	
Maraganore, 2005, USA, REP [C] ‡	–	–	
Maraganore, 2005, USA-LEAPS, GWAS [C] ‡	–	–	
Mueller, 2005, Germany-I [C] • ‡	–	–	
Mueller, 2005, Germany-II [C] • ‡	–	–	

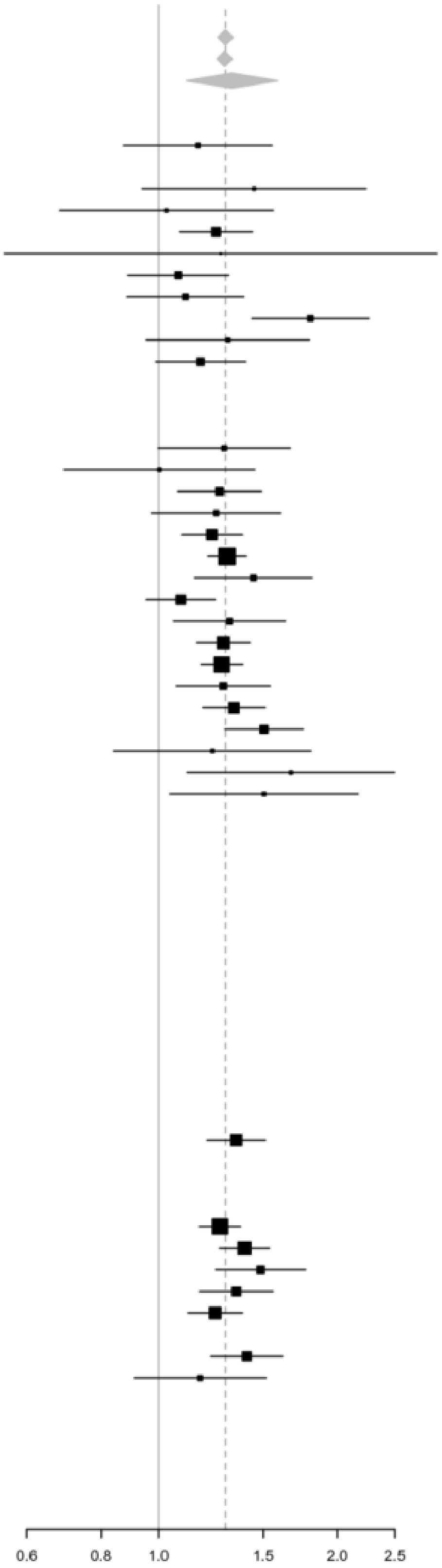

Figure S2, panel 9. *ITGA8* rs7077361: C vs. T

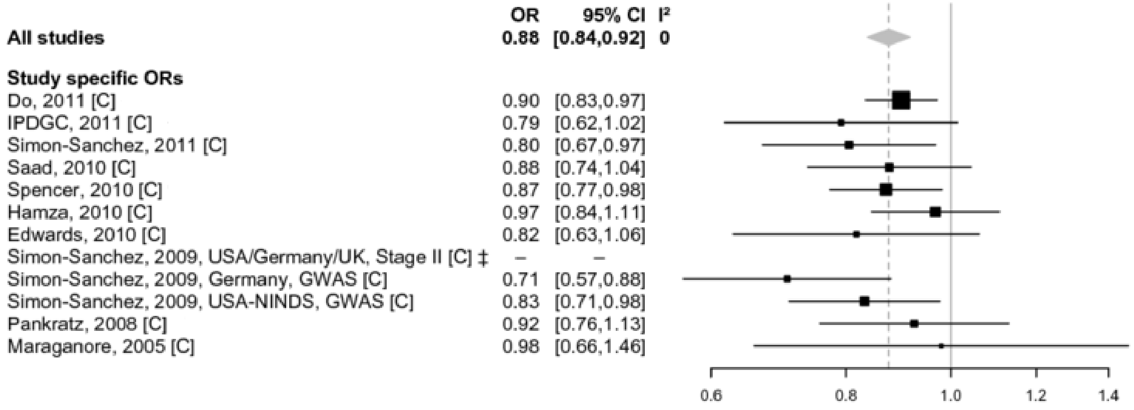

Figure S2, panel 10. *LRRK2* rs1491942: G vs C

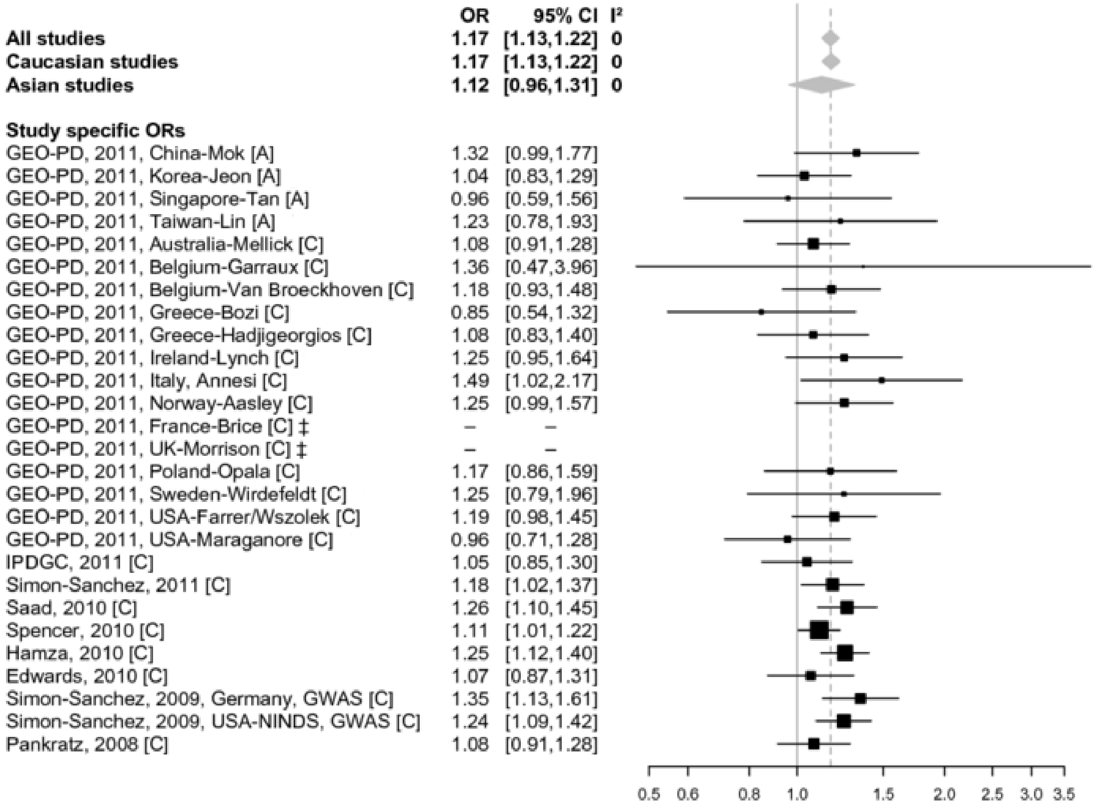

Figure S2, panel 11. *CCDC62/HIP1R* rs10847864: T vs G

	OR	95% CI	I^2
**All studies**	**1.15**	**[1.11,1.18]**	**0**
**Caucasian studies**	**1.15**	**[1.11,1.18]**	**0**
**Asian studies**	**1.12**	**[0.97,1.30]**	**0**
**Study specific ORs**			
GEO-PD, 2011, China-Mok [A]	1.06	[0.80,1.40]	
GEO-PD, 2011, Korea-Jeon [A]	1.13	[0.91,1.40]	
GEO-PD, 2011, Singapore-Tan [A]	1.50	[0.99,2.29]	
GEO-PD, 2011, Taiwan-Lin [A]	0.97	[0.64,1.46]	
GEO-PD, 2011, Australia-Mellick [C]	1.12	[0.97,1.29]	
GEO-PD, 2011, Belgium-Garraux [C]	1.32	[0.56,3.12]	
GEO-PD, 2011, Belgium-Van Broeckhoven [C]	1.08	[0.89,1.31]	
GEO-PD, 2011, Greece-Bozi [C]	1.17	[0.77,1.80]	
GEO-PD, 2011, Greece-Hadjigeorgios [C]	1.13	[0.90,1.42]	
GEO-PD, 2011, Ireland-Lynch [C]	1.06	[0.84,1.33]	
GEO-PD, 2011, Italy, Annesi [C]	1.18	[0.86,1.62]	
GEO-PD, 2011, Norway-Aasley [C]	1.09	[0.91,1.31]	
GEO-PD, 2011, France-Brice [C] ‡	–	–	
GEO-PD, 2011, UK-Morrison [C] ‡	–	–	
GEO-PD, 2011, Poland-Opala [C]	1.13	[0.87,1.46]	
GEO-PD, 2011, Sweden-Wirdefeldt [C]	1.16	[0.78,1.73]	
GEO-PD, 2011, USA-Farrer/Wszolek [C]	1.32	[1.11,1.57]	
GEO-PD, 2011, USA-Maraganore [C]	1.30	[1.01,1.68]	
IPDGC, 2011, France, REP [C]	1.44	[1.15,1.82]	
IPDGC, 2011, Germany, REP [C]	1.14	[0.99,1.31]	
IPDGC, 2011, Netherlands, REP [C]	1.16	[0.93,1.44]	
IPDGC, 2011, UK, REP [C]	1.18	[1.06,1.30]	
IPDGC, 2011, USA, REP [C]	1.10	[1.02,1.20]	
Simon-Sanchez, 2011 [C]	1.07	[0.94,1.21]	
Saad, 2010 [C]	1.22	[1.08,1.37]	
Spencer, 2010 [C]	1.16	[1.06,1.26]	
Simon-Sanchez, 2009, Germany, GWAS [C]	1.30	[1.12,1.51]	
Simon-Sanchez, 2009, USA-NINDS, GWAS [C]	1.02	[0.91,1.15]	
Pankratz, 2008 [C]	1.17	[0.98,1.40]	

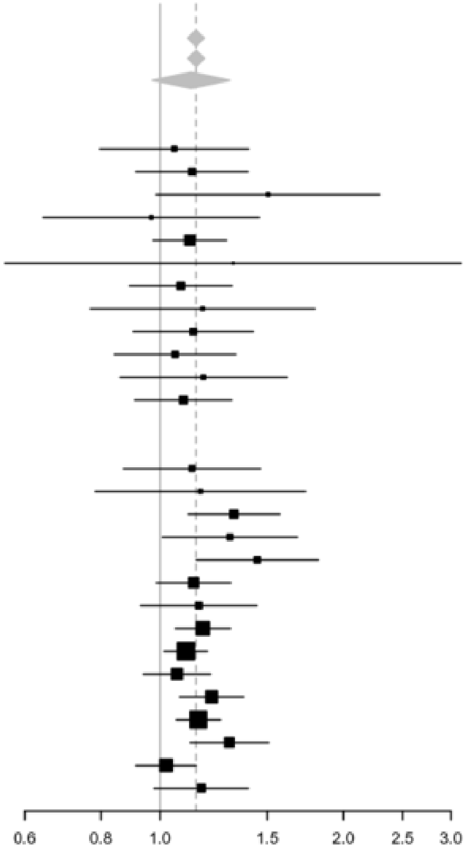

Figure S2, panel 12. *MAPT* H1H2: H2 vs H1

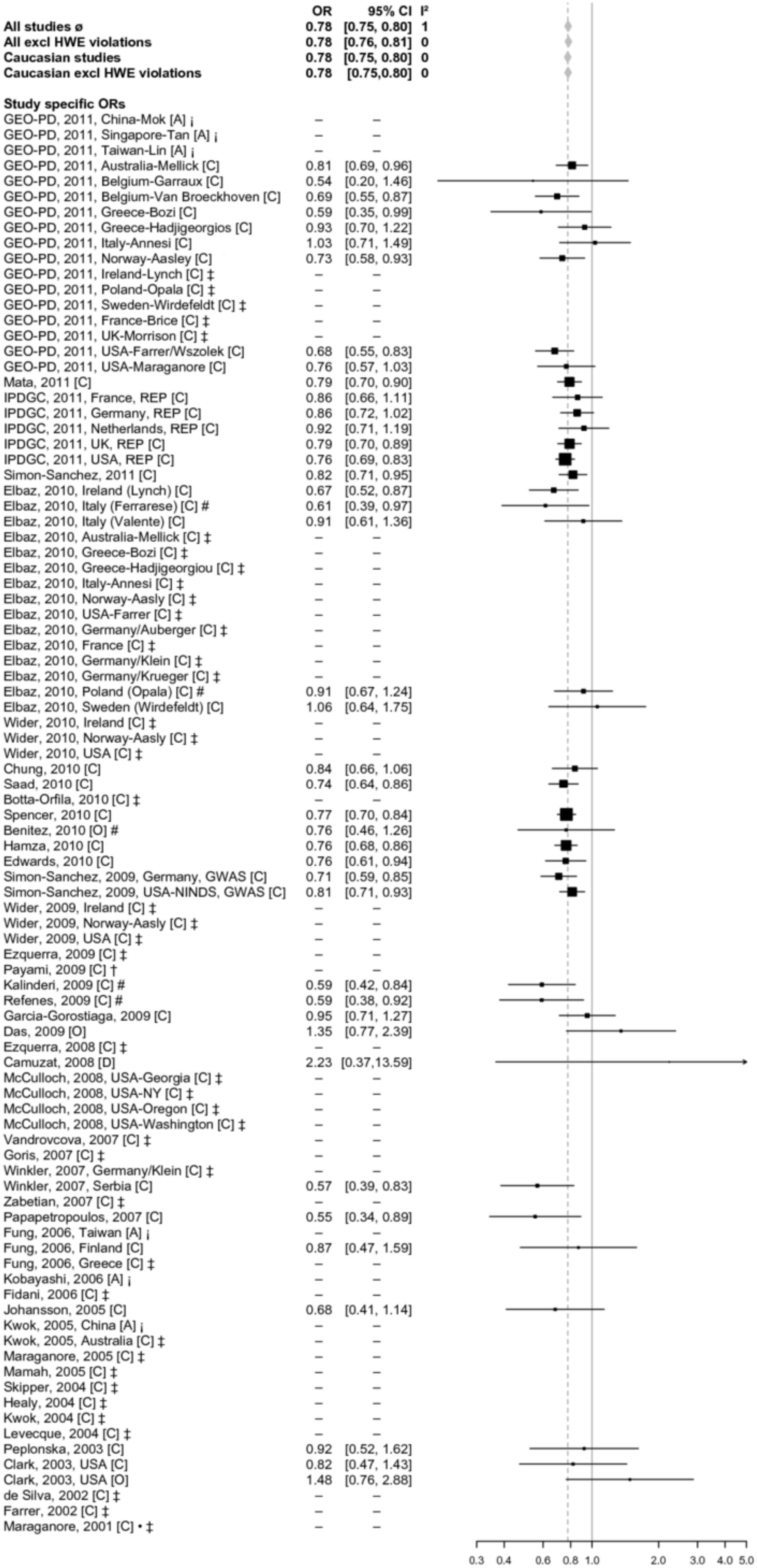

Figure S2, panel 13. PARK16 rs823156: G vs A

	OR	95% CI	I²
**All studies**	**0.86**	**[0.81,0.91]**	**54**
**Caucasian studies**	**0.90**	**[0.85,0.95]**	**41**
**Asian studies**	**0.74**	**[0.68,0.81]**	**0**
**Study specific ORs**			
Mata, 2011 [C]	0.88	[0.75,1.03]	
Do, 2011 [C]	0.83	[0.78,0.88]	
IPDGC, 2011 [C]	0.94	[0.66,1.35]	
Chang, 2011 [A]	0.74	[0.60,0.92]	
Simon-Sanchez, 2011 [C]	0.99	[0.84,1.16]	
Saad, 2010 [C]	0.83	[0.72,0.96]	
Spencer, 2010 [C]	0.98	[0.88,1.08]	
Hamza, 2010 [C]	0.91	[0.80,1.03]	
Tan, 2010 [A]	0.77	[0.63,0.95]	
Edwards, 2010 [C]	1.10	[0.88,1.38]	
Satake, 2009, Japan, Replication-1 [A]	0.80	[0.68,0.94]	
Satake, 2009, Japan, Replication-2 [A]	0.66	[0.51,0.85]	
Satake, 2009, Japan, GWAS [A]	0.72	[0.62,0.83]	
Simon-Sanchez, 2009, USA/Germany/UK, Stage II [C] ‡	–	–	
Simon-Sanchez, 2009, Germany, GWAS [C]	0.88	[0.73,1.06]	
Simon-Sanchez, 2009, USA-NINDS, GWAS [C]	0.83	[0.72,0.95]	
Pankratz, 2008 [C]	0.98	[0.82,1.17]	
Fung, 2006 [C] ‡	–	–	

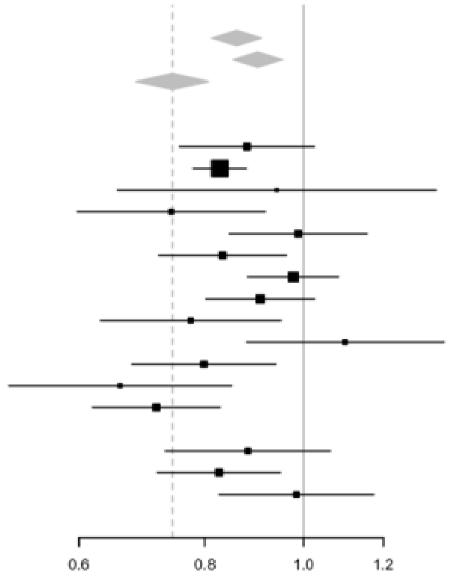

Figure S2, panel 14. *BST1* rs4538475: G vs A

	OR	95% CI	I²
**All studies**	**0.89**	**[0.84,0.93]**	**40**
**All excl initial**	**0.88**	**[0.84,0.92]**	**31**
**Caucasian studies**	**0.91**	**[0.87,0.96]**	**21**
**Caucasian excl initial**	**0.91**	**[0.87,0.94]**	**1**
**Asian studies**	**0.80**	**[0.75,0.86]**	**0**
**Study specific ORs**			
Do, 2011 [C]	0.89	[0.83,0.96]	
IPDGC, 2011 [C]	1.04	[0.86,1.25]	
Simon-Sanchez, 2011 [C]	0.96	[0.83,1.13]	
Saad, 2010 [C]	0.83	[0.71,0.97]	
Spencer, 2010 [C]	0.87	[0.78,0.97]	
Hamza, 2010 [C]	0.90	[0.79,1.02]	
Edwards, 2010 [C]	0.88	[0.69,1.11]	
Satake, 2009, Japan, Replication-1 [A]	0.80	[0.71,0.90]	
Satake, 2009, Japan, Replication-2 [A]	0.82	[0.69,0.97]	
Satake, 2009, Japan, GWAS [A]	0.80	[0.72,0.89]	
Simon-Sanchez, 2009, USA/Germany/UK, Stage II [C] ‡	–	–	
Simon-Sanchez, 2009, Germany, GWAS [C]	0.87	[0.71,1.05]	
Simon-Sanchez, 2009, USA-NINDS, GWAS [C]	1.04	[0.90,1.20]	
Pankratz, 2008 [C]	0.84	[0.70,1.02]	
Fung, 2006 [C] ‡	–	–	
Maraganore, 2005 [C]	1.32	[0.90,1.94]	

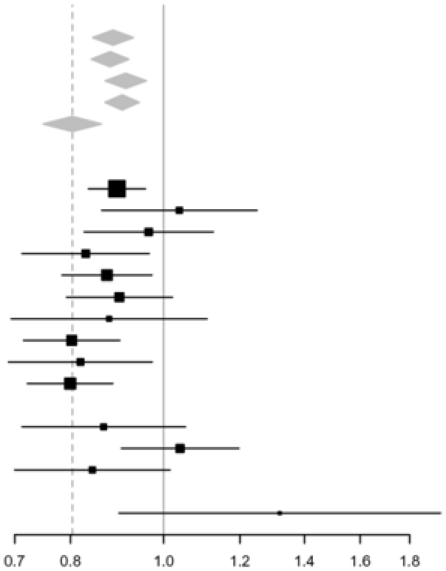

Figure S2, panel 15. *SNCA* rs6532194: T vs C

	OR	95% CI	I²
**All studies**	**1.23**	**[1.14,1.32]**	**45**
**Asian studies**	**1.29**	**[1.20,1.39]**	**31**
**Caucasian studies**	**1.14**	**[1.02,1.27]**	**27**
**Study specific ORs**			
Chang, 2011 [A]	1.27	[1.07,1.50]	
Simon-Sanchez, 2011 [C]	1.08	[0.87,1.34]	
Saad, 2010 [C]	1.22	[1.02,1.46]	
Tan, 2010 [A]	1.26	[1.07,1.48]	
Satake, 2009, Japan (Replication-2) [A]	1.21	[1.01,1.44]	
Satake, 2009, Japan (Replication-1) [A]	1.22	[1.08,1.37]	
Satake, 2009, Japan (GWAS) [A]	1.44	[1.30,1.61]	
Simon-Sanchez, 2009 [C]	1.23	[1.06,1.42]	
Pankratz, 2008 [C]	0.95	[0.75,1.20]	
Fung, 2006 [C] ‡	–	–	

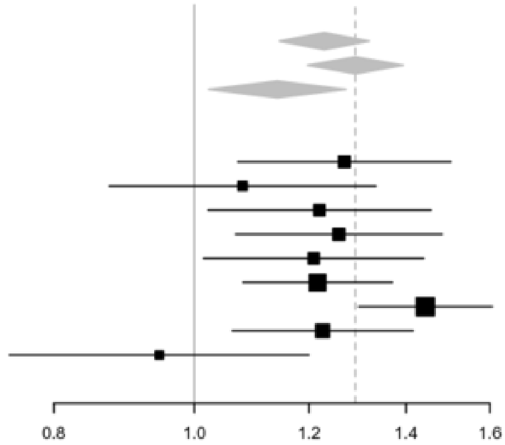

Figure S2, panel 16. *LRKK2* rs34778348: A vs G

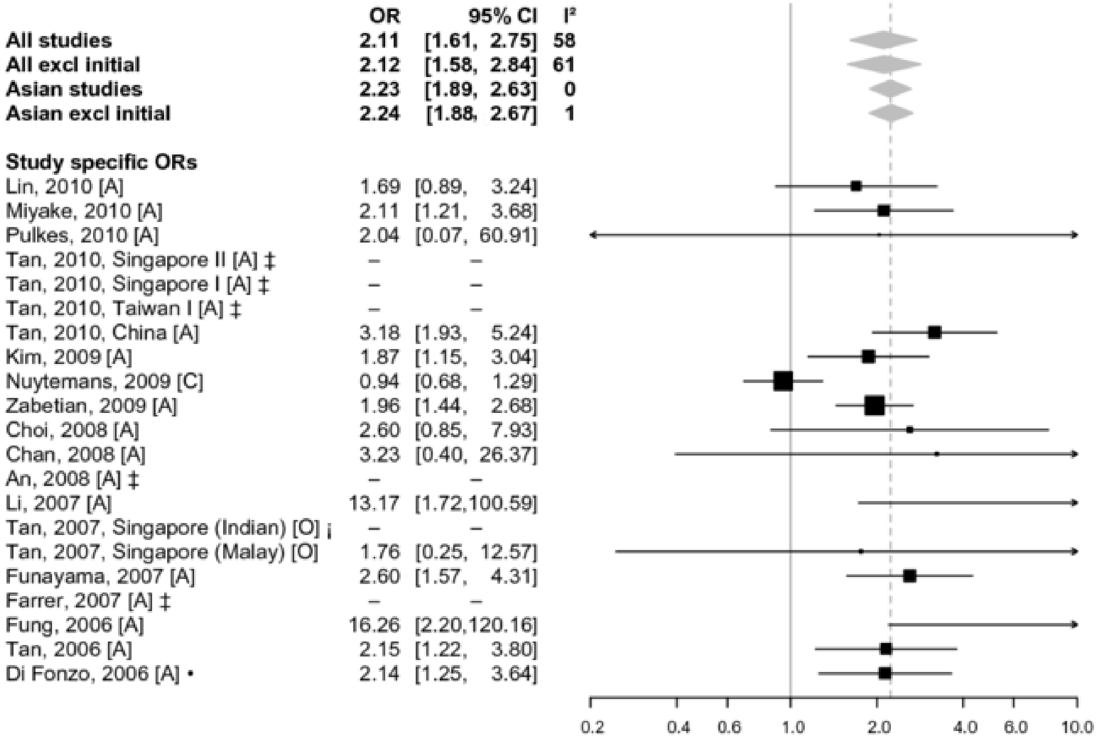

Supplement: Figure S2 — Forest plots of allelic meta-analyses for SNPs showing genome-wide significant association (P<5×10^−8^) with PD susceptibility in the March 31st 2011 datafreeze. Study-specific allelic odds ratios (ORs, black squares) and 95% confidence intervals (CIs, lines) were calculated for each included dataset. The summary OR and CI was calculated using random-effects models (grey diamond). Whenever multiple polymorphisms showed genome-wide significant association in the same locus, only the variant with the smallest P-value is listed here for meta-analysis results after stratification for Caucasian and Asian ancestries. For a complete list of meta-analyses performed for the datafreeze, see Table S1. Figure S1, panel 1-S1, panel 12 and S1, panel 13-S1, panel 16 display the SNP showing the most significant genome-wide association in datasets of Caucasian ancestry and Asian ancestry, respectively. Details and references of all included studies displayed here can be found on the PDGene database (http://www.pdgene.org). I2 = estimate of percentage of between-study heterogeneity that is beyond chance, “excl initial” = summary OR and 95%CI after meta-analysis after exclusion of the initial study, C = Caucasian ancestry, A = Asian ancestry, H = Hispanic descent, D = African descent, “•” = initial study (applies to candidate-gene studies), “†” = no data provided or data was not eligible for inclusion in meta-analysis, “‡” = study excluded due to overlap, “#” = HWE violation in controls (P<0.05, not applicable to quality-controlled GWAS datasets, see Text S1), “i” = SNP monomorphic in the respective dataset, “ø” = meta-analysis after excluding initial study not applicable. (PDF) [file pgen.1002548.s002.pdf]
