# Supplementary material for: Comprehensive Research Synopsis and Systematic Meta-Analyses in Parkinson's Disease Genetics: The PDGene Database
Source: PLoS Genet. 2012 Mar 15;8(3):e1002548. doi: 10.1371/journal.pgen.1002548 (PMC3305333; doi:10.1371/journal.pgen.1002548)
Supplement: Table S2 — Investigation of the extent of statistical inflation assuming sample overlaps of 1%, 5%, and 10% across cases and controls in datasets originating from the same countries. Hypothetical sample overlap across datasets was assumed between different candidate-gene/replication studies and between candidate-gene/replication studies and GWAS datasets if they originated from the same country. These analyses were performed applying random-effects models and adding the sum of weighted co-variances of overlapping datasets to the overall study variance (see ref. [24] in the main text). Note that the assumption of undetected overlapping samples does not apply (and was therefore not modeled here) to overlap between individual GWAS as duplicate samples in these datasets were removed prior to meta-analysis. It also does not apply to independent datasets used in the same publication where duplicate samples had been removed by the authors prior to analysis and publication. We emphasize that this table describes hypothetical scenarios, because the geographical origin of each study had been investigated extensively and potentially overlapping datasets had been excluded as part of PDGene's data inclusion protocol. Thus, the extent of overlap across geographically distinct datasets within the same countries is reduced to accidental recruitment of the same subjects more than once in different datasets throughout the respective countries, and can be expected to be less than ∼1%. This estimate is based on data of the GEO-PD consortium, where sufficient data were centrally available of 6,072 subjects from 20 geographically distinct sites in 13 countries that had been investigated for potentially duplicate samples across sites, but no duplicate subjects (neither between not within countries) were identified when matching on ethnicity, birth, sex, and genotype. The investigation of overlap was not applicable here for Asian datasets, as they originated from different countries and/or were clean [file pgen.1002548.s006.doc]

| **Caucasian ethnicity** | | | | | | | |
| --- | --- | --- | --- | --- | --- | --- | --- |
| **Locus** | **Polymorphism** | **OR** | ***P*-value**  **naive** | **# assumed pairwise overlaps** | ***P*-value**  **1% overlap** | ***P*-value**  **5% overlap** | ***P*-value**  **10% overlap** |
| *GBA* | N370S | 3.51 | 1.44x10^-14^ | 10 | 1.55x10^-14^ | 2.10x10^-14^ | 3.03x10^-14^ |
| *SYT11/RAB25* | chr1:154105678 | 1.73 | 2.35x10^-12^ | 0 | n.a. | n.a. | n.a. |
| PARK16 | rs947211 | 0.91 | 8.00x10^-10^ | 0 | n.a. | n.a. | n.a. |
| *STK39* | rs2390669 | 1.19 | 1.37x10^-09^ | 3 | 3.02x10^-08^ | 3.58x10^-08^ | 4.41x10^-08^ |
| *MCCC1/LAMP3* | rs11711441 | 0.86 | 9.20x10^-10^ | 13 | 1.02x10^-09^ | 1.50x10^-09^ | 2.38x10^-09^ |
| *DGKQ* | rs11248060 | 1.21 | 3.04x10^-12^ | 0 | n.a. | n.a. | n.a. |
| *BST1* | rs11724635 | 0.88 | 1.87x10^-10^ | 16 | 2.09x10^-10^ | 2.94x10^-10^ | 4.39x10^-10^ |
| *SNCA* | rs356219 | 1.29 | 6.06x10^-65^ | 22 | 1.57x10^-64^ | 6.41x10^-63^ | 5.02x10^-61^ |
| *ITGA8* | rs7077361 | 0.88 | 1.51x10^-08^ | 0 | n.a. | n.a. | n.a. |
| *LRRK2* | rs1491942 | 1.17 | 6.44x10^-15^ | 11 | 7.57x10^-15^ | 1.47x10^-14^ | 3.22x10^-14^ |
| *CCDC62/HIP1R* | rs10847864 | 1.15 | 4.37x10^-17^ | 9 | 5.04x10^-17^ | 8.94x10^-17^ | 1.77x10^-16^ |
| *MAPT/STH* | H1H2 | 0.78 | 7.97x10^-52^ | 45 | 2.04x10^-51^ | 1.90x10^-49^ | 3.32x10^-47^ |
