# Supplementary material for: Comprehensive Research Synopsis and Systematic Meta-Analyses in Parkinson's Disease Genetics: The PDGene Database
Source: PLoS Genet. 2012 Mar 15;8(3):e1002548. doi: 10.1371/journal.pgen.1002548 (PMC3305333; doi:10.1371/journal.pgen.1002548)
Supplement: Text S1 — Supplementary material. This file includes supplementary methods and references as well as the list of members of the GWAS consortia, the GEO-PD Consortium, and consortia-specific acknowledgements. (PDF) [file pgen.1002548.s007.pdf]

Comprehensive research synopsis and systematic meta-analyses in Parkinson's disease genetics: The PDGene database

Christina M. Lill, Johannes T. Roehr, Matthew B. McQueen,
Fotini K. Kavvoura, Sachin Bagade, Brit-Maren M. Schjeide, Leif M. Schjeide,
Esther Meissner, Ute Zauft, Nicole C. Allen, Tian Liu, Marcel Schilling,
Kari J. Anderson, Gary Beecham, Daniela Berg, Joanna M. Biernacka, Alexis Brice,
Anita L. DeStefano, Chuong B. Do, Nicholas Eriksson, Stewart A. Factor, Matthew J. Farrer,
Tatiana Foroud, Thomas Gasser, Taye Hamza, John A. Hardy, Peter Heutink, Erin Hill-Burns,
Christine Klein, Jeanne C. Latourelle, Demetrius M. Maraganore, Eden R. Martin, Maria Martinez,
Richard H. Myers, Michael A. Nalls, Nathan Pankratz, Haydeh Payami, Wataru Satake,
William K. Scott, Manu Sharma, Andrew B. Singleton, Kari Stefansson, Tatsushi Toda,
Joyce Y. Tung, Jeffery Vance, Nick W. Wood, Cyrus P. Zabetian, 23andMe Inc.,
The Genetic Epidemiology of Parkinson's Disease Consortium (GEO-PD),
The International Parkinson's Disease Genomics Consortium (IPDGC),
The Parkinson's Disease GWAS Consortium,
The Wellcome Trust Case Control Consortium 2 (WTCCC2),
Peter Young, Rudolph E. Tanzi, Muin J. Khoury, Frauke Zipp, Hans Lehrach,
John P. A. Ioannidis, Lars Bertram

Text S1 (Supplementary material)

pages	content
2-6	Supplementary Methods
7-13	Members of the GWAS consortia, the GEO-PD Consortium, and consortia-specific acknowledgements
14-15	Supplementary References

Supplementary Methods

Literature Searches

Inclusion criteria: For inclusion in PDGene, a study has to meet three criteria:
1) It must evaluate the association between a bi-allelic genetic polymorphism (minor allele frequency [MAF] ≥0.01 in the healthy control population of at least one study) and Parkinson's disease (PD) risk in datasets comprised of both affected (defined as clinically and/or neuropathologically diagnosed "Parkinson's disease") and unaffected individuals.
2) It must be published in a peer-reviewed journal. This criterion specifically excludes studies reported only in abstract format, e.g. at scientific meetings.
3) The study must be published in English.

Exclusion criteria: While studies using family-based methods were included in the qualitative gene summary pages on PDGene, genotype distributions were excluded from the statistical analyses (except when raw genotype data were available, which was applicable for the LEAPS GWAS) [1]. Of note, case-control studies that were enriched for family history were not excluded. Genetic association studies published in non-English studies were excluded due to practical reasons. The percentage of published non-English genetic association studies in PD is small, and more than half of these non-English papers are also published in an English language journal (and hence included in PDGene if there were otherwise eligible). For instance, an exploratory Pubmed search for all PD genetic association papers published between 2009 and 2010 yielded a total of two potentially eligible, non-English publications [2,3] (out of 177), thus representing ~1% of all studies published during this time period. Furthermore, based on the content of the abstracts, the same authors had previously reported identical or largely overlapping results in an English journal [4,5]. Studies examining genotype-phenotype correlations in PD subjects only (but not examining "healthy" controls), or studies exclusively using PD dementia as their "case" definition were not included. Whenever a sample contains patients suffering from both PD with and without dementia, only data on PD subjects without dementia were utilized if they were given separately for this group. Studies using only "diseased" controls, e.g. hospital-based controls suffering from other diseases, were excluded. Studies on polymorphisms with three or more alleles were usually excluded from the meta-analyses. Proxy SNPs, i.e. SNPs tagging other variants, were generally not combined in the meta-analysis due to potentially different linkage disequilibrium patterns across different populations and ethnicities. An exception was made for the *MAPT* H1H2 haplotype, where frequencies were determined based on tagging-variants (mostly SNPs) as described in the original publications. However, this excluded tagging microsatellites. Variants in mitochondrial DNA tested in association studies were included in the qualitative gene summaries on PDGene but were not subjected to meta-analysis because of the multicopy nature of the mitochondrial genome, and the high frequency of somatic mutation events that vary substantially across tissues [6], which may introduce a potential bias into the meta-analyses. Studies using a "pooled DNA approach" were included in the qualitative gene summary pages on PDGene, but data were excluded from the PDGene meta-analyses. For exclusion criteria of data from analyses due to errors in study conduct or presentation, please see the section about additional quality control below.

Search strategies: To identify potential association studies eligible for inclusion in PDGene, we performed a PubMed search using the terms "parkinson* AND associat*" before January 1st 2007, and performed daily searches for "parkinson*" thereafter until March 31st 2011. This search yielded 27,210 articles, which were screened for eligibility using the title, abstract, or full-paper, as necessary. Additional screening of bibliographies in reviews, published meta-analyses, and original genetic association studies were also performed. Overall, full text versions of 1,534 articles were obtained. Following the inclusion and exclusion criteria outlined above, 828 articles were included in PDGene until March 31st 2011 (also see Figure 1).

Data Management

Demographic variables: Details extracted for each included study consisted of the first author name, year of publication, and PubMed-identifier, along with key population-specific details extracted from each study (if provided), such as ascertainment design (family-based or case-control), ethnic background and population (i.e. country) of origin, as well as source (clinic-, or population-based) of the samples, the number of cases with gender ratio, age at onset, age at examination, and method of diagnosis (see below), the number of controls with gender ratio and age at examination, and the reported study results. All data were first entered into a local, offline database, after which all entries were double-checked by an independent member of our group against the original publications.

Genetic association data: Whenever possible, "dbSNP" identifiers ("rs-ID") were used to designate polymorphism identity throughout the database. If these were not specified in the original articles, we attempted to resolve rs-IDs using bioinformatics tools such as NCBI's "BLAST" (http://www.ncbi.nlm.nih.gov/BLAST/) and Ensembl (http://www.ensembl.org/index.html), which allowed us to determine rs-IDs for ~80% of all polymorphisms initially not published with an rs-ID. If an rs-ID

could not be determined unequivocally, we adopted the most commonly used nomenclature of the primary publications. Genotype distributions or published allelic odds ratios (ORs) and confidence intervals (CIs) were extracted for each polymorphism from the publications where provided. Published allelic ORs and CIs were used if they were calculated either without adjustment for covariates, or if they had been adjusted for age and/or sex and/or for population substructure, and/or corrected for genomic inflation; effect size estimates based on adjustment for other covariates were not included. In cases where allele frequencies, but not genotype distributions or allelic ORs, were presented in a publication, genotype distributions were calculated from reported allele frequencies and sample sizes (assuming no violation of Hardy Weinberg equilibrium [HWE] unless reported otherwise in the original paper). First and last authors of studies with missing genetic association data were generally contacted twice via e-mail and asked to supply their genotype/allele distributions for the investigated polymorphisms.

Additional quality control of genetic association data: Potential errors in study conduct and reporting of results (e.g. erroneous genotyping/sequencing protocols, genotype/allele counts that did not match the effective sample size provided in the demographic description of the respective publications, allele mismatches, erroneous rs-identifiers, etc.) were carefully investigated by cross-checking of relevant data extracted from the publications, and, where applicable, by automated comparison to public databases (NCBI's dbSNP [http://www.ncbi.nlm.nih.gov/sites/entrez], UCSC Genome Browser [http://genome.ucsc.edu/]). Deviations from allele frequencies in controls of more than 10% in comparison to ethnicity-specific allele frequency data of the International HapMap Consortium and 1000G were flagged as a potential error. Upon identification of these and other potential errors, the authors of the respective publications were generally contacted for clarification. In rare events, the discrepancies could not be resolved, the study was included in the qualitative overview pages on PDGene, but data were excluded from the meta-analyses. For studies reporting association results on polymorphisms without providing NCBI's rs-identifiers or applying non-commercial, custom-made genotyping and sequencing protocols, the genotyping protocol was carefully re-evaluated by comparison to the appropriate human reference sequence, and, as outlined above, the investigated variant was assigned an existing rs-identifier, if available.

Duplicate publications: In many cases, authors report the same association results in identical or largely overlapping samples in separately published articles. Whenever such overlap between two (or more) studies was evident by information provided in the original publication, or suspected overlap was confirmed by the authors, duplicate or overlapping datasets were excluded from the meta-analyses. Where possible the dataset with the largest sample size was included in the meta-analysis. Data on additional polymorphisms provided in the overlapping studies that were not investigated in the other study were still included in the respective meta-analyses.

Large-scale association studies. The extent of data integration from large-scale genotyping studies (e.g. GWAS) was based on the availability of the data. Whenever possible, individual-level data was obtained (via NCBI's "dbGaP" database), and study-specific ORs determined after data-cleaning and adjustment for age, sex and population stratification (outlined below). In cases where only summary-level genotype data were available (e.g. the extended "NINDS/Germany" sample, see below), we used the allelic ORs and 95% CIs supplied by the original investigators. If neither individual-level or summary-level genome-wide genotype data were available, we extracted as much of the data as possible from the primary publications. Generally, preference was given to include allelic ORs and CIs adjusted for population stratification as reported in the primary publications. If these were not available or not eligible, crude ORs were calculated from the provided genotype/allele summary data.

Statistical Analyses:

Statistical analysis for this manuscript were performed in SAS v9.2, the PLINK toolset v1.07 [7] and the R programming language, version 2.10.0, using packages HardyWeinberg, version 1.4 and rmeta, version 2.16, with the exception of the GWAS-only meta-analyses (see below).

Meta-analyses: For all variants with minor allele frequencies>0.01 in at least one control population meeting our inclusion criteria (see above) crude study-specific ORs and 95% CIs were calculated for each dataset using allelic contrasts (usually comparing minor vs. major allele). For studies with no available genotype or allele distributions, we used allelic ORs and CIs as given in the original publication where provided. Random-effects allelic meta-analyses [8] were performed if a minimum of four independent datasets existed per polymorphism. This procedure was first performed on all studies irrespective of ethnicity. Summary ORs and 95% CIs were also calculated for studies of distinct ethnic groups, i.e. Caucasians, and Asians, if three or more such studies exist.

Sensitivity analyses and between-study heterogeneity: Sensitivity analysis entailed calculating summary ORs and 95% CIs for all studies after excluding the initial report, and after excluding smaller-scale studies that violated HWE according to a chi-square test implemented in the "HardyWeinberg" R package at $p \leq 0.05$. In case of cell counts below 5 Fisher's exact test was used instead of the chi-square test. Between-study heterogeneity was assessed by calculating the I^2 heterogeneity metric. I^2 is estimated based on the Q

statistic (Q-(df)/Q), and it takes values from 0 to 100% that show the extent of the heterogeneity that is beyond chance. In contrast to Q, which cannot be compared across meta-analyses with different numbers of studies, I^2 is comparable regardless of the number of studies meta-analyzed. Generally values above 50% are considered to represent large heterogeneity [9,10]. When there are only few studies, both Q and I^2 carry considerable uncertainty and should be interpreted cautiously [11].

Evaluating the credibility of significant associations: Each nominally significant meta-analysis result was assessed based on two different approaches:

1) Calculation of Bayes factors (BF): BFs were calculated assuming an average non-null odds ratio of 1.15 as approximation of a typical “complex disease effect size” based on a spike and smear prior distribution of effects [12], and expressed as the ratio of the probability of H1 vs. H0. That is, the higher the BF, the higher the odds that H1 is true vs. H0, while a BF of 1 suggests that both hypotheses are equally likely. BFs are expressed as $\log_{10}$(BF) (abbreviated as "logBF") throughout our paper. Generally, a logBF≥5, which suggests that the data increase over 100,000-fold the odds of a given finding being true vs. not true, is considered as "sufficiently convincing" support in the context of genetic association studies [13].

2) Grading based on Human Genome Epidemiology Network (HuGENet) interim criteria for the assessment of cumulative evidence of genetic associations [14,15]. These criteria take into account "amount of evidence" (i.e. sample size, measured as "N minor"), "consistency of replication" (i.e. heterogeneity across studies, measured as "I^2"), and "protection from bias" ("Bias reason"). For amount of evidence, grade A is when the total number of minor alleles of cases and controls combined in the meta-analyses exceeds 1000, B when it is between 100 and 1000, and C when it is less than 100. Consistency of replication, was assessed by determining the I^2 heterogeneity metric (see above). I^2 point estimates<25% obtain grade A, values of 25-50% are rated grade B, and I^2 exceeding 50% is rated C. This criterion does not apply to meta-analyses with a *P*-value<$1x10^{-7}$ after exclusion of the initial studie(s), as described in Khoury et al, 2009 [15]. For protection from bias, the following potential reasons for bias in the meta-analysis results are systematically assessed: summary OR<1.15 ("Low OR"), loss of significance after exclusion of first study ("F", see above), loss of significance after exclusion of studies violating HWE in control populations ("HWE", see above), evidence for publication/small-study bias using a modified regression test ("Regr") [16]. If none of the latter conditions are met grade "A" is assigned, otherwise grade "C". This criterion does not apply to meta-analyses with a *P*-value<$1x10^{-7}$ (including all data for prospective analyses, e.g. GWAS, and after exclusion of the initial studie(s) for retrospective analyses), as described in Khoury et al, 2009 [15]. The overall epidemiological credibility is graded as "A" (=strong) if associations received three A grades, "B" (=moderate) if they received at least one B grade but no C grades, and "C" (=weak) if they received a C grade in any of the three assessment criteria.

GWAS-only meta-analyses

Datasets: Three independent sets of individual-level and/or summary genotype data were obtained via the dbGaP repository (http://www.ncbi.nlm.nih.gov/gap) and used in the GWAS meta-analyses. The **first dataset** contained individual-level genotype data from the "Mayo-Perlegen Linked Efforts to Accelerate Parkinson’s Solutions (hereafter referred to as "LEAPS") Collaboration" (phs000048.v1.p1; health research consent; embargo release March 3, 2008) [1]. Genotyping was performed using a Perlegen platform (198,345 SNPs). This family-based dataset consists of 443 discordant sibling pairs (DSP; prior to data cleaning), such that each sibling pair has one member affected with PD and one unaffected. **The second dataset** is part of the National Institute of Neurological Disorders and Stroke-Genome-Wide Genotyping in Parkinson’s Disease (hereafter referred to as "NINDS") [17]. Genotyping was performed using HumanHap550 BeadChips (Illumina, Inc.). For this study, individual-level genotype data for subjects from the United States were available on dbGaP under accession phs000089.v3.p2 ("Individual genotype-calls of common SNP loci"; consent group: general research use; embargo release: April 09, 2010) for 545,066 SNPs in a total of 940 PD cases and 801 controls (prior to data cleaning). This dataset was used for genome-wide genotype imputation after data cleaning (see below). The investigators also released genotype summary data for 463,185 SNPs in a larger sample comprising 1,713 PD cases and 3,978 controls from the United States and Germany (hereafter referred to as "NINDS/Germany"; accession pha002868.1 [Analysis data files for associations between genotype and phenotype variables; consent group: general research use; embargo release: January 11, 2010]) [17]. Quality control filtering for these data was performed by the GWAS investigators prior to release of the data. Since this latter sample was considerably larger than phs000089.v3.p2, we used the ORs provided by the GWAS investigators in the GWAS meta-analyses where applicable, i.e. for SNPs with data in the US-only and US/Germany datasets, results from the latter were used. The **third dataset** contained individual-level genotype data from the “Parkinson’s Research: The Organized Genetics Initiative (PROGENI)” and “Genome-wide scan for Parkinson's disease” (GenePD) Initiative (hereafter referred to as "PROGENI/GenePD", phs000126.v1.p1; general research use and Parkinson’s Disease and related disorders consent groups, embargo release February 13, 2009) [18]. Genotypes from the Illumina HumanCNV 370v1 (344,301 SNPs) were available on 900 PD cases and 867 controls (prior to data cleaning).

Data Cleaning: Data cleaning was performed with the PLINK toolset v1.07 [7]. Duplicate and related samples: Prior to any analysis, we excluded samples found to overlap or to represent either 1st or 2nd degree relatives within or across the NINDS (n=19 samples) and PROGENI/GenePD (n=10 samples) datasets (provided by Drs. N. Pankratz and T. Foroud). Subsequent genome-wide pairwise IBS estimation on the combined NINDS and PROGENI/GenePD datasets confirmed that no sample pairs existed in the resulting dataset with PI-HAT values>0.2. No overlapping or related samples were found between LEAPS and the other two datasets. Genotyping quality: The remaining samples were cleaned on the basis of genotyping efficiency and quality by excluding all SNPs with a MAF<0.01, missing rates>2%, or HWE violations with *P*-values<$1x10^{-6}$. In addition, individual samples with genotyping efficiency<95% were excluded. This resulted in 149,817 (across 433 PD and 428 controls), 508,861 (931 PD, 790 controls), and 328,529 (888 PD, 857 controls) analyzable SNPs from the LEAPS, NINDS, PROGENI/GenePD datasets, respectively. Outlier detection and population stratification: For the two case-control datasets (NINDS, PROGENI/GenePD) we used genome-wide pairwise IBS estimations to determine outlying samples, and multidimensional scaling (MDS) to create a set of MDS co-variates for adjustment for population stratification in the subsequent genetic association analyses. First, we determined a set of low-LD SNPs from each GWAS dataset (using the "indep-pairwise" command in PLINK on windows of 100 SNPs, a stepsize of 5 and maximum r^2 threshold of 0.2). This resulted in 109,114 and 86,516 SNPs for the NINDS and PROGENI/GenePD datasets, respectively. Second, we determined outlying samples by identifying the five nearest neighbors of each sample based on pairwise IBS distances (using the "neighbor" command in PLINK) and excluded samples deviating -4 or more standard deviations, i.e. 23 and 9 samples in the NINDS and PROGENI/GenePD datasets, respectively. Third, we extracted MDS dimensions from the genome-wide pairwise IBS distances of the remaining samples (using the "mds-plot" option in PLINK). The first two MDS dimensions from these analyses were used to account for population stratification in the subsequent association analyses (see below).

Genome-wide genotype imputations: Genotypes for uncovered autosomal SNPs were inferred from the cleaned LEAPS, NINDS, and PROGENI/GenePD datasets using the IMPUTE program v2.0 [19]. Before imputation, all genotypes were recoded to reflect alignment to the "plus" strand using information provided along with the GWAS datasets from dbGaP. As reference for the imputations, we used the precompiled "HapMap 3 + 1000GP CEU+TSI" panels from the IMPUTE website (obtained on June 2nd, 2010). These contain HapMap 3 data (from release #2, Feb 2009) and 1,000 Genomes Project data from Pilot 1 genotypes (released in August 2009) for autosomal SNPs. In the two case-control datasets (NINDS, PROGENI/GenePD) only SNPs with an average maximum posterior genotype call probability across all samples (i.e. a "certainty" score) ≥0.4 and an estimated minor allele frequency of 0.01 were considered further. In the family-based dataset (LEAPS), individual-level genotypes were assigned according to the genotype called with 0.9 or greater posterior call probability, or coded as missing if the posterior call probability fell below 0.9. Next, imputed genotyped datasets were cleaned following the same thresholds as outlined above (i.e. exclusion of SNPs with MAF<0.01, HWE *P*-value<$1x10^{-6}$, genotyping efficiency<0.98, and exclusion of samples with average missing genotype rates>5%). Overall, this led to final datasets of 735,746 SNPs (in 431 PD and 427 controls; LEAPS), 7,050,115 SNPs (in 905 PD and 782 controls; NINDS), and 7,319,987 SNPs (in 861 PD and 852 controls; PROGENI/GenePD). **Within-Study Association Analyses:** Association analyses were performed in SAS v9.2 for the LEAPS and SNPTEST v2 [19] for the NINDS and PROGENI/GenePD datasets. For the family-based dataset (LEAPS) we used a conditional logistic regression approach stratifying on family to estimate the additive (copies of minor allele) OR for autosomal SNPs [20]. For the case-control datasets (NINDS and PROGENI/GenePD), we used the frequentist under an additive transmission model adjusted for age, sex and population stratification (using the first two MDS dimensions) and an EM model to adjust for genotype uncertainty following imputation to estimate sample-specific ORs for each SNP. For the NINDS dataset, 463,185 quality cleaned SNPs were released as summary data for an extended dataset ("NINDS/Germany", see above). Since this extended dataset was substantially larger than the sample released with individual-level genotype data, we used the reported ORs for these SNPs in the subsequent meta-analyses.

Meta-Analyses of GWAS results: Meta-analyses based on random-effects and fixed-effect models were performed in PLINK v1.07 [7] using the study-specific ORs and standard errors (SE's; scaled by the square root of each sample's genomic inflation factor) calculated as outlined above. Overall, there were a total of 7,723,931 unique SNPs, 7,123,920 of which were present in at least two, 711,271 in at least three datasets. In addition, we used the METAL software [21] (Aug 2010 version) to perform P-value based meta-analyses weighted by sample size. Both methods led to comparable results although the random-effects meta-analyses tended to be more conservative. The genomic-inflation factor did not exceed 1.007 after removal of the established PD susceptibility genes *SNCA*, *LRRK2*, *MAPT*, and *DGKQ*/*GAK* (see Figure S1).

Additional data
For 147 SNPs of the 867 PDGene core meta-analyses yielding P-values of ≤0.1, we obtained additional summary data from all remaining Caucasian GWAS datasets. For the established loci *SNCA*, *MAPT*, and *LRRK2* only the SNPs reported by the recent GWAS meta-analysis [22] were requested. In addition, we obtained data from the newly identified SNP rs7077361 in *ITGA8* from the Japanese GWAS dataset [23]. Furthermore, we obtained summary data for ten SNPs reported in the recent GWAS meta-analysis [22] generated in datasets of the GEO-PD Consortium [24]. Details on sample characteristics, data cleaning, and, if applicable, imputation protocols as well as statistical analyses can be found in the individual publications [22–30] . Briefly, data of the following additional datasets were provided to us by investigators from the respective sites:
1) 23andMe [25]**:** 112 SNPs in 3,426 PD cases and 29,624 controls;
2) UK (WTCCC2) [26]**:** 140 SNPs in 1,705 PD cases and 5,175 controls, genotyped on the Illumina Human660-Quad platform (cases) and on the Illumina 1.2M Duo platform (controls);
3) USA (NGRC) [27]**:** 135 SNPs in 1,956 PD cases and 1,982 controls, genotyped on the Illumina HumanOmni1-Quad platform;
4) France [28]**:** 140 SNPs in 1,039 PD cases and 1,984 controls, genotyped on the Illumina Human610-Quad platform;
5) Netherlands [29]**:** 138 SNPs in 824 PD cases and 2,082 controls, genotyped on the Illumina Human660W-Quad platform (cases);
6) Iceland [22]**:** 124 SNPs in 479 PD cases and 1,427 controls, genotyped on Illumina Bead Arrays;
7) Germany [17]**:** 138 SNPs in 742 PD cases and 944 controls, genotyped on Illumina 550Kv1 arrays; and the Human610K platform (controls);
8) USA (HIHG) [30]**:** 120 SNPs in 579 PD cases and 619 controls, genotyped on the Illumina Human610-Quad platform (cases),and the Illumina Human610-Quad, Illumina HumanHap550, and 1M-Duo Infinium HD BeadChip platforms (controls).
9) GEO-PD [24]**:** 10 SNPs in 5,702 PD cases and 4,973 controls of Caucasian descent (excluding DNA samples from the UK and France overlapping with the GWAS datasets used by the recent GWAS-meta-analysis [22]), and 1,022 PD cases and 874 controls of Asian descent.
10) Japan [23]**:** 1 SNP in 988 PD cases and 2,521 controls, genotyped on Illumina HumanHap550 platforms.

Online Database Structure
PDGene database. After completion of the data entering, processing and analyses described in detail above, all study-specific variables, genotype data (except for GWAS), and meta-analysis plots are posted on a dedicated, publicly available, online adaptation of the PDGene database using the same software and code as our databases for Alzheimer's disease [31] and schizophrenia [32]. The online database is hosted by the "Alzheimer Research Forum", a non-profit, internet-based community portal dedicated to furthering collaboration among researchers to help in the search for causes, treatments, and understanding of Alzheimer's disease. The PDGene site can be accessed via its own designated URL (http://www.pdgene.org).
Summaries of large-scale studies: In addition to systematically including association results from GWAS, large-scale studies are summarized in a dedicated section on PDGene. This section distinguishes between "GWAS" (≥100,000 independent markers) and "Other large-scale association studies" (≥1,000 independent genetic markers; this latter section also includes re-analyses or meta-analyses of previous GWAS). In addition to providing the main characteristics of each study, this section also provides a hyperlinked list of "featured genes", i.e. those loci or pathways highlighted by the primary authors as the main outcome of their study after having completed all analyses. Note that the criteria used to define of "featured genes" varies across publications.
Display of meta-analysis results on UCSC Genome Browser. All meta-analysis results were compressed into "BigWig" format (i.e. indexed binary files) [33] displayed as custom track on the UCSC Genome Browser (URL: http://genome.ucsc.edu/cgi-bin/hgTracks?org=human&db=hg18&position=chr4&hgt.customText=http://xgene.molgen.mpg.de/ucsc/pdgenetracks.txt). This feature plots random-effects meta-analysis $-\log_{10}$ *P*-values (on the y-axis), against genomic location (based on build NCBI36/hg18; on the x-axis). This includes the results of all 7,123,920 GWAS meta-analyses as well as the results of all 867 meta-analyses included in PDGene proper. Whenever SNPs from the GWAS meta-analyses overlapped with those from PDGene, *P*-values from the latter were used for display as these were always based on larger sample sizes. SNP names were based on dbSNP build 131, transcribed to build NCBI36/hg18.

Members of the GWAS consortia and the GEO-PD Consortium

Members of the IPDGC
Vincent Plagnol[1], Michael A Nalls[2], Jose M Bras[3], Dena G Hernandez[2,3], Manu Sharma[5], Una-Marie Sheerin[3], Mohamad Saad[5,6], Javier Simón-Sánchez[7], Claudia Schulte[4], Suzanne Lesage[8,9,10], Sigurlaug Sveinbjörnsdóttir[11,12,13], Philippe Amouyel[51,52], Sampath Arepalli[1], Gavin Band[66], Roger A Barker[16], Céline Bellinguez[66], Yoav Ben-Shlomo[17], Henk W Berendse[18], Daniela Berg[4], Kailash Bhatia[19], Rob MA de Bie[20], Alessandro Biffi[21,22,23], Bas Bloem[24], Zoltan Bochdanovits[7], Michael Bonin[25], Kathrin Brockmann[4], Janet Brooks[1], David J Burn[26], Gavin Charlesworth[3], Honglei Chen[27], Patrick F Chinnery[28], Sean Chong[2], Carl E Clarke[29,30], Mark R Cookson[2], J Mark Cooper[31], Jean Christophe Corvol[8,9,10,32], Carl Counsell[33], Philippe Damier[34], Jean-François Dartigues[35], Panos Deloukas[36], Günther Deuschl[37], David T Dexter[38], Karin D van Dijk[18], Allissa Dillman[2], Frank Durif[39], Alexandra Dürr[8,9,10,15], Sarah Edkins[36], Jonathan R Evans[40], Thomas Foltynie[41], Colin Freeman[66], Jianjun Gao[27], Michelle Gardner[3], Raphael Gibbs[2,3], Alison Goate[42], Emma Gray[36], Rita Guerreiro[3], Ómar Gústafsson[14], Clare Harris[33], Garrett Hellenthal[66], Jacobus J van Hilten[43], Albert Hofman[44], Albert Hollenbeck[45], Janice Holton[46], Michele Hu[47], Xuemei Huang[48], Heiko Huber[4], Gavin Hudson[28], Sarah E Hunt[36], Johanna Huttenlocher[14], Thomas Illig [49], Pálmi V Jónsson[50], Cordelia Langford[40], Andrew Lees[46], Peter Lichtner[53], Patricia Limousin[54], Grisel Lopez[55], Delia Lorenz[37], Alisdair McNeill[31], Catriona Moorby[29], Matthew Moore[2], Huw Morris[56], Karen E Morrison[29,57], Ese Mudanohwo[58], Sean S O'Sullivan[46], Justin Pearson[56], Richard Pearson[66], Joel S Perlmutter[42], Hjörvar Pétursson[14,25], Matti Pirinen[66], Pierre Pollak[59], Bart Post[24], Simon Potter[36], Bernard Ravina[60], Tamas Revesz[46], Olaf Riess[25], Fernando Rivadeneira[44,61], Patrizia Rizzu[7], Mina Ryten[3], Stephen Sawcer[62], Anthony Schapira[31], Hans Scheffer[63], Karen Shaw[46], Ira Shoulson[64], Ellen Sidransky[55], Rohan de Silva[3], Colin Smith[65], Chris C A Spencer[66], Hreinn Stefánsson[14], Stacy Steinberg[14], Joanna D Stockton[29], Amy Strange[66], Zhan Su[66], Kevin Talbot[67], Carlie M Tanner[68], Avazeh Tashakkori-Ghanbaria[36], François Tison[69], Daniah Trabzuni[3], Bryan J Traynor[2], André G Uitterlinden[44,61], Jana Vandrovcova[3], Daan Velseboer[20], Marie Vidailhet[8,9,10], Damjan Vukcevic[66], Robert Walker[65], Bart van de Warrenburg[24], Michael E Weale[72], Mirdhu Wickremaratchi[70], Nigel Williams[56], Caroline H Williams-Gray[16], Sophie Winder-Rhodes[71], Kári Stefánsson[14], Maria Martinez[5,6], Peter Donnelly[66], John Hardy[3], Peter Heutink[7], Alexis Brice[8,9,10,15], Thomas Gasser[4], Nicholas W Wood[1,3], Andrew B Singleton[2]

1UCL Genetics Institute, London, UK. 2Laboratory of Neurogenetics. National Institute on Aging, National Institutes of Health, Bethesda, MD, USA. 3Department of Molecular Neuroscience, UCL Institute of Neurology, London, UK. 4Department for Neurodegenerative Diseases, Hertie Institute for Clinical Brain Research, University of Tübingen, and DZNE, German Center for Neurodegenerative Diseases, Tübingen, Germany. 5INSERM UMR 1043, CPTP, Toulouse, France. 6Paul Sabatier University, Toulouse, France. 7Department of Clinical Genetics, Section of Medical Genomics, VU University Medical Centre, Amsterdam, The Netherlands. 8INSERM, UMR_S975, Paris, France. 9Université Pierre et Marie Curie-Paris, Centre de Recherche de l'Institut du Cerveau et de la Moelle épinière, UMR-S975, Paris, France. 10CNRS, UMR 7225, Paris, France. 11Department of Neurology, Landspítali University Hospital, Reykjavík, Iceland. 12Department of Neurology, MEHT Broomfield Hospital, Chelmsford, Essex, UK. 13Queen Mary College, University of London, London, UK. 14deCODE genetics, Reykjavik, Iceland. 15AP-HP, Pitié-Salpêtrière Hospital, Department of Genetics and Cytogenetics, Paris, France. 16Department of Neurology, Addenbrooke's Hospital, University of Cambridge, Hills Road, Cambridge, UK. 17Department of Social Medicine, Bristol University, UK. 18Department of Neurology and Alzheimer Center, VU University Medical Center, Amsterdam, The Netherlands. 19Department of Motor Neuroscience, UCL Institute of Neurology, Queen Square, London, UK. 20Department of Neurology, Academic Medical Center, University of Amsterdam, Amsterdam, The Netherlands. 21Center for Human Genetic Research, Massachusetts General Hospital, Boston, MA, USA. 22Department of Neurology, Massachusetts General Hospital, Boston, MA, USA. 23Program in Medical and Population Genetics, Broad Institute, Cambridge, MA, USA. 24Department of Neurology, Radboud University Nijmegen Medical Centre, Nijmegen, The Netherlands. 25Department of Medical Genetics, Institute of Human Genetics, University of Tübingen, Tübingen, Germany. 26Newcastle University Clinical Ageing Research Unit, Campus for Ageing and Vitality Newcastle upon Tyne, UK. 27Epidemiology Branch, National Institute of Environmental Health Sciences, National Institutes of Health, NC, USA. 28Neurology M4104, The Medical School, Newcastle upon Tyne, UK. 29School of Clinical and Experimental Medicine, University of Birmingham, Edgbaston, Birmingham, UK. 30Department of Neurology, City Hospital, Sandwell and West Birmingham Hospitals NHS Trust, Birmingham, UK. 31Department of Clinical Neurosciences, UCL Institute of Neurology, Royal Free Campus, London UK. 32INSERM CIC-9503, Hôpital Pitié-Salpêtrière, Paris, France. 33University of Aberdeen, Division of Applied Health Sciences, Population Health Section, Polwarth building, Foresterhill, Aberdeen, UK. 34CHU Nantes, CIC0004, Service de Neurologie, Nantes, France. 35INSERM U897, Université Victor Segalen, Bordeaux, France. 36Wellcome Trust Sanger Institute, Wellcome Trust Genome Campus, Hinxton, Cambridge, UK. 37Klinik

für Neurologie, Universitätsklinikum Schleswig-Holstein, Campus Kiel, Christian-Albrechts-Universität Kiel, Kiel, Germany. 38Parkinson's Disease Research Group, Faculty of Medicine, Imperial College London, Hammersmith Hospital, London, UK. 39Service de Neurologie, Hôpital Gabriel Montpied, Clermont-Ferrand, France. 40Cambridge Centre for Brain Repair, Forvie Site, Robinson Way, Cambridge, UK. 41Institute of Neurology, University College London, London, UK. 42Department of Psychiatry, Department of Neurology, Washington University School of Medicine, St. Louis, MO, USA. 43Department of Neurology, Leiden University Medical Center, Leiden, The Netherlands. 44Department of Epidemiology, Erasmus University Medical Center, Rotterdam, The Netherlands. 45AARP, Washington DC, USA 46Queen Square Brain Bank for Neurological Disorders, Institute of Neurology, University College London, London, UK. 47Department of Clinical Neurology, John Radcliffe Hospital, Oxford, UK. 48Departments of Neurology, Radiology, Neurosurgery, Pharmacology, Kinesiology & Bioengineering, Pennsylvania State University-Milton S. Hershey Medical Center, Hershey, PA, USA. 49Institute of Epidemiology, Helmholtz Zentrum München, German Research Centre for Environmental Health, Neuherberg, Germany. 50Department of Geriatrics, Landspítali University Hospital, Reykjavík, Iceland. 51INSERM U744, Lille, France. 52Institut Pasteur de Lille, Université de Lille Nord, Lille, France. 53Institute of Human Genetics, Helmholtz Zentrum München, German Research Centre for Environmental Health, Neuherberg, Germany. 54Institute of Neurology, Sobell Department, Unit of Functional Neurosurgery, Queen Square, London, UK. 55Section on Molecular Neurogenetics, Medical Genetics Branch, NHGRI, National Institutes of Health, Bethesda, MD, USA. 56MRC Centre for Neuropsychiatric Genetics and Genomics, Cardiff University School of Medicine, Cardiff, UK. 57Neurosciences Department, Queen Elizabeth Hospital, University Hospitals Birmingham NHS Foundation Trust, Birmingham, UK. 58Neurogenetics Unit, UCL Institute of Neurology/National Hospital for Neurology and Neurosurgery, London, UK. 59Service de Neurologie, CHU de Grenoble, Grenoble, France. 60Translational Neurology, Biogen Idec, Cambridge, MA, USA. 61Department of Internal Medicine, Erasmus Medical Center, Rotterdam, The Netherlands. 62University of Cambridge, Department of Clinical Neurosciences, Addenbrooke's hospital, Cambridge, UK. 63Department of Human Genetics, Radboud University Nijmegen Medical Centre, Nijmegen, The Netherlands. 64Department of Neurology, University of Rochester, Rochester, NY, USA. 65Department of Pathology, Wilkie Building, Teviot Place, Edinburgh, UK. 66Wellcome Trust Centre for Human Genetics, Oxford, UK. 67University of Oxford, Department of Clinical Neurology, John Radcliffe Hospital, Oxford, UK. 68Clinical Research Department, The Parkinson's Institute and Clinical Center, Sunnyvale, CA, USA. 69Service de Neurologie, Hôpital Haut-Lévêque, Pessac, France. 70Department of Neurology, Cardiff University, Cardiff, UK. 71Department of Psychiatry and Medical Research Council/Wellcome Trust Behavioural and Clinical Neurosciences Institute, University of Cambridge, Cambridge, UK. 72 King's College London, Department of Medical and Molecular Genetics, London UK.

The contributors of the WTCCC2

Peter Donnelly (Chair)[1,2], Ines Barroso (Deputy Chair)[3], Jenefer M Blackwell[4,5], Elvira Bramon[6], Matthew A Brown[7], Juan P Casas[8], Aiden Corvin[9], Panos Deloukas[3], Audrey Duncanson[10], Janusz Jankowski[11], Hugh S Markus[12], Christopher G Mathew[13], Colin N A Palmer[14], Robert Plomin[15], Anna Rautanen[1], Stephen J Sawcer[16], Richard C Trembath[13], Ananth C Viswanathan[17], Nicholas W Wood[18], Chris C A Spencer[1], Gavin Band1, Céline Bellenguez[1], Colin Freeman[1], Garrett Hellenthal[1], Eleni Giannoulatou[1], Matti Pirinen[1], Richard Pearson[1], Amy Strange[1], Zhan Su[1], Damjan Vukcevic[1], Peter Donnelly[1,2], Cordelia Langford[3], Sarah E Hunt[3], Sarah Edkins[3], Rhian Gwilliam[3], Hannah Blackburn[3], Suzannah J Bumpstead[3], Serge Dronov[3], Matthew Gillman[3], Emma Gray[3], Naomi Hammond[3], Alagurevathi Jayakumar[3], Owen T McCann[3], Jennifer Liddle[3], Simon C Potter[3], Radhi Ravindrarajah[3], Michelle Ricketts[3], Matthew Waller[3], Paul Weston[3], Sara Widaa[3], Pamela Whittaker[3], Ines Barroso[3], Panos Deloukas[3], Mark I McCarthy[19]

1Wellcome Trust Centre for Human Genetics, Oxford, UK. 2Dept Statistics, University of Oxford, Oxford, UK. 3Wellcome Trust Sanger Institute, Wellcome Trust Genome Campus, Hinxton, Cambridge, UK. 4Telethon Institute for Child Health Research, Centre for Child Health Research, University of Western Australia, Western Australia, Australia. 5Cambridge Institute for Medical Research, University of Cambridge School of Clinical Medicine, Cambridge, UK.
6Department of Psychosis Studies, NIHR Biomedical Research Centre for Mental Health at the Institute of Psychiatry, King's College London and The South London and Maudsley NHS Foundation Trust, London, UK. 7Diamantina Institute of Cancer, Immunology and Metabolic Medicine, Princess Alexandra Hospital, University of Queensland, Brisbane, Queensland, Australia. 8Dept Epidemiology and Population Health, London School of Hygiene and Tropical Medicine, London, and Dept Epidemiology and Public Health, University College London, UK. 9Neuropsychiatric Genetics Research Group, Institute of Molecular Medicine, Trinity College Dublin, Dublin, Ireland. 10Molecular and Physiological Sciences, The Wellcome Trust, London, UK. 11Centre for Digestive Diseases, Queen Mary University of London, London E1 2AD, UK and Digestive Diseases Centre, Leicester Royal Infirmary, Leicester, UK and

Department of Clinical Pharmacology, Old Road Campus, University of Oxford, Oxford, UK. 12Clinical Neurosciences, St George's University of London, London, UK. 13King's College London Dept Medical and Molecular Genetics, School of Medicine, Guy's Hospital, London, UK. 14Biomedical Research Centre, Ninewells Hospital and Medical School, Dundee, UK. 15King's College London Social, Genetic and Developmental Psychiatry Centre, Institute of Psychiatry, Denmark Hill, London, UK. 16University of Cambridge Dept Clinical Neurosciences, Addenbrooke's Hospital, Cambridge, UK. 17NIHR Biomedical Research Centre for Ophthalmology, Moorfields Eye Hospital NHS Foundation Trust and UCL Institute of Ophthalmology, London, UK. 18Dept Molecular Neuroscience, Institute of Neurology, Queen Square, London, UK. 19Oxford Centre for Diabetes, Endocrinology and Metabolism (ICDEM), Churchill Hospital, Oxford, UK.

Funding/Acknowledgments IPDGC/WTCCC2

This work was supported in part by the Wellcome Trust/MRC Joint Call in Neurodegeneration award (WT089698) to the UK Parkinson's Disease Consortium (UKPDC) whose members are from the UCL/Institute of Neurology, the University of Sheffield and the MRC Protein Phosphorylation Unit at the University of Dundee. Additionally part of the study was undertaken at UCLH/UCL using funding through a Department of Health NIHR Biomedical Research Centre. This work was also supported by Parkinson's UK (Grants 8047 and J-0804) and the Medical Research Council (G0700943). Genotyping of UK replication cases on Immunochip was part of the Wellcome Trust Case Control Consortium 2 project which is funded by the Wellcome Trust (085475/B/08/Z and 085475/Z/08/Z).

JH receives support from the Reta Lila Weston Trust for Medical Research. This work was also supported by the Landspitali University Hospital Research Fund (S.Sv.), the Icelandic Research Council (S.Sv.), the European Community Framework Programme 7, People programme, IAPP on novel genetic and phenotypic markers of Parkinson's disease and Essential Tremor (MarkMD), contract no PIAP-GA-2008-230596 MarkMD. (HP, JH).

This US work was supported in part by the Intramural Research Programs of the National Institute on Aging, National Institute of Neurological Disorders and Stroke, National Institute of Environmental Health Sciences, National Human Genome Research Institute, National Institutes of Health, Department of Health and Human Services; project numbers Z01 AG000949-02 and Z01-ES101986. In addition this study was supported by the US Department of Defense, award number W81XWH-09-2-0128. Funding to support collection of a portion of the samples was obtained from the National Institutes of Health (grants NS057105 and RR024992), the American Parkinson Disease Association (APDA), Barnes Jewish Hospital Foundation and the Greater St Louis Chapter of the APDA.

The KORA research platform (KORA: Cooperative Research in the Region of Augsburg; http://www.gsf.de/KORA) was initiated and financed by the Forschungszentrum für Umwelt und Gesundheit (GSF), which is funded by the German Federal Ministry of Education, Science, Research and Technology and by the State of Bavaria. The study was additionally funded by the German National Genome Network (NGFNplus #01GS08134; German Ministry for Education and Research) and in addition by the German Federal Ministry of Education and Research (BMBF) NGFN (01GR0468) and in the frame of ERA-Net NEURON (01GW0908). This work was also supported by the Helmholtz Alliance Mental Health in an Ageing Society (HelMA, HA-215) funded by the Initiative and Networking Fund of the Helmholtz Association.

The French GWA scan work was supported by the French National Agency of Research (http://www.agence-nationale-recherche.fr <http://www.agence-nationale-recherche.fr> , ANR-08-MNP-012) and by the National Research Funding Agency (ANR-08-NEUR-004-01) in ERA-NET NEURON framework (http://www.neuron-eranet.eu).

We also want to thank the Hersenstichting Nederland (http://www.hersenstichting.nl <http://www.hersenstichting.nl>), the Neuroscience Campus Amsterdam and the section of Medical genomics, the Prinses Beatrix Fonds (http://www.prinsesbeatrixfonds.nl <http://www.prinsesbeatrixfonds.nl>) for sponsoring this work.

This study utilized the high-performance computational capabilities of the Biowulf Linux cluster at the National Institutes of Health, Bethesda, Md. (http://biowulf.nih.gov <http://biowulf.nih.gov>). DNA panels and samples from the NINDS Human Genetics Resource Center DNA and Cell Line Repository (http://ccr.coriell.org/ninds) were used in this study, as well as clinical data. The submitters that contributed samples are acknowledged in detailed descriptions of each panel (http://ccr.coriell.org/sections/Collections/NINDS/?SsId=10 <http://ccr.coriell.org/sections/Collections/NINDS/?SsId=10>).

The authors thank The French Parkinson's Disease Genetics Study Group: Y. Agid, M. Anheim, A.-M. Bonnet, M. Borg, A. Brice, E. Broussolle, J.-C. Corvol, Ph. Damier, A. Destée, A. Dürr, F. Durif, S. Klebe, E. Lohmann, M. Martinez, P. Pollak, O. Rascol, F. Tison, C. Tranchant, M. Vérin, F. Viallet and M. Vidailhet. The authors thank the members of the French 3C consortium: Drs Annick Alpérovitch, Claudine Berr, Christophe Tzourio and Jean-Charles Lambert for giving us the possibility to use part of the 3C

cohort; and Drs. M Lathrop and D Zelenika for their support in generating the genome-wide molecular data.
UK population control data was made available through WTCCC1. We thank Jeffrey Barrett for assistance with the design of the Immunochip.

The Parkinson's Disease GWAS Consortium
PSG-PROGENI Investigators and Coordinators: Albany Medical College: S Factor, D Higgins, S Evans; Barrow Neurological Institute: H Shill, M Stacy, J Danielson, L Marlor, K Williamson; Baylor College of Medicine: J Jankovic, C Hunter; Beth Israel Deaconess Medical Center: D Simon, P Ryan, L Scollins; Beth Israel Medical Center: R Saunders-Pullman, K Boyar, C Costan-Toth, E Ohmann; Brigham & Women's Hospital: L Sudarsky, C Joubert; Brown University (Memorial Hospital of RI): J Friedman, K Chou, H Fernandez, M Lannon; Cleveland Clinic Florida-Weston: N Galvez-Jimenez, A Podichetty, K Thompson; Clinical Neuroscience Center: P Lewitt, M DeAngelis; Colorado Neurological Institute: C O'Brien, L Seeberger, C Dingmann, D Judd; Columbia University Medical Center: K Marder, J Fraser, J Harris; Creighton University: J Bertoni, C Peterson; Evanston Northwestern Healthcare: M Rezak, G Medalle; Hotel-Dieu Hospital-Chum: S Chouinard, M Panisset, J Hall, H Poiffaut; Hunter Homes McGuire Veterans Medical Center: V Calabrese, P Roberge; Indiana University School of Medicine: J Wojcieszek, J Belden; Institute For Neurodegenerative Disorders: D Jennings, K Marek, S Mendick; Johns Hopkins University: S Reich, B Dunlop; London Health Sciences Centre: M Jog, C Horn; Mayo Clinic Jacksonville: R Uitti, M Turk; McFarland Neurosciences: T Ajax, J Mannetter; Medical College of Georgia: K Sethi, J Carpenter, B Dill, L Hatch, K Ligon, S Narayan; Medical College of Wisconsin: K Blindauer, K Abou-Samra, J Petit; Medical University of Ohio: L Elmer, E Aiken, K Davis, C Schell, S Wilson; Mount Sinai School of Medicine: M Velickovic, W Koller (deceased), S Phipps; North Shore-LIJ Health System: A Feigin, M Gordon, J Hamann, E Licari, M Marotta-Kollarus, B Shannon, R Winnick; Northwestern University: T Simuni, A Videnovic, A Kaczmarek, K Williams, M Wolff; Ochsner Clinic Foundation: J Rao, M Cook; Ohio State University: M Fernandez, S Kostyk, J Hubble, A Campbell, C Reider, A Seward; Oregon Health & Science University: R Camicioli, J Carter, J Nutt, P Andrews, S Morehouse, C Stone; Ottawa Hospital Civic Site: T Mendis, D Grimes, C Alcorn-Costa, P Gray, K Haas, J Vendette; Pacific Neuroscience Medical Group: J Sutton, B Hutchinson, J Young; Saskatoon Dist Health Board Royal Univ Hosp: A Rajput, A Rajput, L Klassen, T Shirley; Scott & White Hospital/Texas A&M University: B Manyam, P Simpson, J Whetteckey, B Wulbrecht; The Parkinson's & Movement Disorder Institute: D Truong, M Pathak, K Frei, N Luong, T Tra, A Tran, J Vo; Toronto Western Hospital, University Health: A Lang, G Kleiner-Fisman, A Nieves, L Johnston, J So; UMDNJ-School of Osteopathic Medicine: G Podskalny, L Giffin; University of Alabama at Birmingham: P Atchison, C Allen; University of Alberta: W Martin, M Wieler; University of Calgary: O Suchowersky, S Furtado, M Klimek; University of California Irvine: N Hermanowicz, S Niswonger; University of California San Diego: C Shults (deceased), D Fontaine; University of California San Francisco: M Aminoff, C Christine, M Diminno, J Hevezi; University of Chicago: A Dalvi, U Kang, J Richman, S Uy, J Young; University of Cincinnati: A Dalvi, A Sahay, M Gartner, D Schwieterman; University of Colorado Health Sciences Center: D Hall, M Leehey, S Culver, T Derian; University of Connecticut: T Demarcaida, S Thurlow; University of Iowa: R Rodnitzky, J Dobson; University of Kansas Medical Center: K Lyons, R Pahwa, T Gales, S Thomas; University of Maryland School of Medicine: L Shulman, S Reich, W Weiner, K Dustin; University of Miami: K Lyons, C Singer, W Koller (deceased), W Weiner, L Zelaya; University of Minnesota: P Tuite, V Hagen, S Rolandelli, R Schacherer, J Kosowicz; University of New Mexico: P Gordon, J Werner; University of Puerto Rico School of Medicine: C Serrano, S Roque; University of Rochester: R Kurlan, D Berry, I Gardiner; University of South Florida: R Hauser, J Sanchez-Ramos, T Zesiewicz, H Delgado, K Price, P Rodriguez, S Wolfrath; University of Tennessee Health Science Center: R Pfeiffer, L Davis, B Pfeiffer; University of Texas Southwestern Medical Center: R Dewey, B Hayward, A Johnson, M Meacham, B Estes; Wake Forest University School of Medicine: F Walker, V Hunt, C O'Neill; Washington University: B Racette, L Good, M Rundle

GenePD Investigators and Coordinators: University Southern California School of Medicine: M Lew; University of Calgary: O Suchowersky; University of Lübeck, Germany: C Klein; UMDNJ-Robert Wood Johnson Medical School: L Golbe, MH Mark; Massachusetts General Hospital, Harvard Medical School: J Growdon, N Huggins; University of Virginia Health System: GF Wooten; University of Alabama at Birmingham: R Watts; University of Toronto: M Guttman; Washington University School of Medicine: B Racette, J Perlmutter; Barrow Neurological Institute: L Marlor; Sun Health Research Institute: H Shill; University of Miami: C Singer; Parkinson Institute, Istituti Clinici di Perfezionamento, Milano, Italy: S Goldwurm, G Pezzoli; Boston University School of Medicine: MH Saint-Hilaire, T Massood; Cleveland Clinic Foundation: K Baker, I Itin; University of Louisville School of Medicine: I Litvan; University of Sydney ANZAC Research Institute, Concord Hospital, Sydney, Australia: G Nicholson, A Corbett; Struthers Parkinson's Center, Minneapolis: M Nance; Port City Neurology, Scarborough, ME: E Drasby;

Parkinson's Disease and Movement Disorder Center of Boca Raton: S Isaacson; Newcastle University, Newcastle upon Tyne, UK: D Burn, P Chinnery; General Regional Hospital Bolzano, Bolzano, Italy: P Pramstaller; University of Arkansas for Medical Sciences: J Al-hinti; Aarhus University Hospital, Aarhus, Denmark: A Moller, K Ostergaard; University of Arizona: S Sherman; Auckland City Hospital, Auckland, New Zealand: R Roxburgh, B Snow; University of Kentucky College of Medicine: J Slevin, F Cambi.

HIHG Investigators and Coordinators: CA Jauregui, MA Nance, RL Watts, JP Hubble, WC Koller, K Lyons, R Pahwa, MB Stern, A Colcher, BC Hiner, J Jankovic, WG Ondo, FH Allen, Jr., CG Goetz, GW Small, D Masterman, F Mastaglia, BL Scott, C Singer, F Nahab, MA Pericak-Vance, and JL Haines who contributed to this study. Some of the samples used in this study were collected while the Udall PDRCE was at Duke University.

Members of the GEO-PD consortium
Australia: From the Queensland Parkinson's Project: R.S Boyle and A Sellbach (Proncess Alexandra Hospital, Brisbane), J.D. O'Sullivan (Royal Brisbane and Women's Hospital Brisbane), George Mellick PhD (Griffith University, Nathan, Queensland, Australia), G.T. Sutherland, G.A Siebert and N.N.W Dissanayaka (Eskitis Institute for Cell and Molecular Therapies, Griffith University, Nathan, QLD). *Belgium:* Christine Van Broeckhoven PhD DSc (Neurodegenerative Brain Diseases Group, Department of Molecular Genetics, VIB; and Laboratory of Neurogenetics, Institute Born-Bunge; and University of Antwerp, Antwerpen), Jessie Theuns, PhD (Neurodegenerative Brain Diseases Group, Department of Molecular Genetics, VIB; and Laboratory of Neurogenetics, Institute Born-Bunge, and University of Antwerp, Antwerpen), David Crosiers MD (Neurodegenerative Brain Diseases Group, Department of Molecular Genetics, VIB; and Laboratory of Neurobiology, Institute Born-Bunge; and University of Antwerp; and Division of Neurology, University Hospital Antwerp, Antwerpen), Barbara Pickut, MD (Memory Clinic and Division of Neurology, ZNA Middelheim, Antwerpen; and Division of Neurology, University Hospital Antwerp, Antwerpen), Sebastiaan Engelborghs, MD, PhD (Laboratory of Neurochemistry and Behavior, Institute Born-Bunge; and University of Antwerp; and Memory Clinic and Division of Neurology, ZNA Middelheim, Antwerpen), Bram Meeus (Neurodegenerative Brain Diseases Group, Department of Molecular Genetics, VIB; and Laboratory of Neurogenetics, Institute Born-Bunge; and University of Antwerp, Antwerpen), Peter P. De Deyn, MD, PhD (Laboratory of Neurochemistry and Behavior, Institute Born-Bunge; and University of Antwerp; and Memory Clinic and Division of Neurology, ZNA Middelheim, Antwerpen), Patrick Cras, MD, PhD (Laboratory of Neurobiology, Institute Born-Bunge; and University of Antwerp; and Division of Neurology, University Hospital Antwerp, Antwerpen), Gaëtan Garraux, MD (Department of Neurology, University Hospital of Liege, Belgium). Funding: This research was in part supported by the Special Research Fund of the University of Antwerp, the Fund for Scientific Research Flanders (FWO-V), the Institute for the promotion of Innovation by Science and Technology in Flanders (IWT-V), the Foundation for Alzheimer Research (SAO/FRMA), the Interuniversity Attraction Poles (IAP) Program P6/43 of the Belgian Federal Science Policy Office, a Methusalem Excellence Grant of the Flanders Government and the Medical Research Foundation Antwerp and Neurosearch, Antwerp,Belgium, and the Alzheimer's Association USA,. B.M. is a holder of a PhD fellowship of the IWT-V, DC is a holder of an FWO-V PhD fellowship and J.T. receives a FWO-V postdoctoral fellowship, Belgium *Brazil:* Renato P. Munhoz, MD (Paraná Parkinson's Disease Foundation, Curitiba/PR, Brazil) *Canada:* Ekaterina Rogaeva PhD (Tanz Centre for Research in Neurodegenerative Diseases, Department of Medicine, Division of Neurology, University of Toronto, ON, Canada); Anthony E Lang, MD, Movement Disorders Centre, Toronto Western Hospital, University of Toronto, Toronto, ON, Canada, Matthew J. Farrer PhD (University of British Columbia, Vancouver, BC, Canada). *Chile:* Luis Lay-Son MD (University of Santiago de Chile, Santiago, Chile) *France:* From the French Parkinson's Disease Genetics Study Group: Y Agid, M Anheim, A-M Bonnet, M Borg, A Brice, E Broussolle, JC Corvol, P Damier, A Destée, A Dürr, F Durif, S Lesage, E Lohmann, P Pollak, O Rascol, F Tison, C Tranchant, F Viallet, M Vidailhet. Also, Alexis Elbaz (Institut National de la Sante et de la Recherche Medicale Unit 975, Universite Pierre et Marie Curie, Paris, France), Alexis Brice MD (Institut National de la Sante et de la Recherche Medicale Unit 975, Universite Pierre et Marie Curie, Paris, France), Christophe Tzourio (Inserm U708, Paris), Philippe Amouyel (Inserm U744, Lille), Marie-Anne Loriot (Inserm UMRS775, Paris). Eugénie Mutez (Inserm UMR837, Service de Neurologie et de Pathologie du Mouvement CHRU de Lille, Univ Lille Nord de France) Aurélie Duflot, (UMR837 Inserm-Univ Lille 2, CHRU de Lille), Jean-Philippe Legendre (Service de Neurologie et Pathologie du Mouvement, Clinique de Neurologie du CHU de Lille), Nawal Waucquier (Service de Neurologie et Pathologie du Mouvement, Clinique de Neurologie du CHU de Lille), the 2 Centres de Ressources Biologiques (IPL-Lille, CHRU-Lille) and its scientific committee (AD,MCCH, P Amouyel, F Pasquier, R Bordet). Funding : This work is supported by the French National Agency of Research (ANR-08-MNP-012). Lille: Financial support from CHRU de Lille, Univ Lille 2, Inserm, French Ministry PHRCs (1994/, 2002/1918, 2005/1914), Association France Parkinson (2005), Fondation de France 2004-013306, Fondation de la Recherche Médicale (2006),

PPF (synucléothèque 2005-2009), *Germany:* Thomas Gasser MD, (Department of Neurology, University Hospital Tuebingen) Olaf Riess MD (Department of Neurology, University Hospital Tuebingen) Daniela Berg MD, (Department of Neurology, University Hospital Tuebingen), Claudia Schulte MSc (Department of Neurology, University Hospital Tuebingen), Christine Klein, MD (Section of Clinical and Molecular Neurogenetics at the Department of Neurology, University of Lübeck), Ana Djarmati, PhD (Department of Neurology, University of Lübeck), Rejko Krüger MD (Center of Neurology and Hertie-Institute for Clinical Brain Research, Tübingen, Germany), Johann Hagenah, MD (Department of Neurology, University of Lübeck), Katja Lohmann, PhD (Section of Clinical and Molecular Neurogenetics at the Department of Neurology, University of Lübeck), Georg Auburger MD (Department of Neurology, Goethe University Frankfurt am Main, Germany), Rüdiger Hilker MD Department of Neurology, Goethe University Frankfurt am Main, Germany), Simone van de Loo MD (Department of Neurology, Goethe University Frankfurt am Main, Germany), Lars Bertram MD (Max Planck Institute, Berlin, Germany). *Greece:* Maria Bozi MD (General Hospital of Syros, Syros, Greece), Efthimios Dardiotis, MD (Department of Neurology, Faculty of Medicine, University of Thessaly and Institute of Biomedical Research & Technology, CERETETH, Larissa), George Hadjigeorgiou, PhD (University of Thessaly Medical School, Larissa, Greece), Vaia Tsimourtou, Styliani Ralli, Persa Kountra, MD, Gianna Patramani, MD, and Cristina Vogiatzi, MD (all: Department of Neurology, Faculty of Medicine, University of Thessaly, Larissa), Katerina Markopoulou MD, PhD (University of Thessaly Medical School, Larissa, Greece), Leonidas Stefanis MD, PhD (University of Athens Medical School, Athens, Greece). *Japan*: Nobutaka Hattori, MD, PhD (Department of Neurology, Juntendo University School of Medicine, Tokyo), Hideshi Kawakami, MD, PhD (University of Hiroshima, Hiroshima, Japan), Hiroyuki Tomiyama, MD, PhD (Department of Neurology, Juntendo University School of Medicine, Tokyo), Manabu Funayama, PhD (Department of Neurology, and Research Institute for Diseases of Old Age, Graduate School of Medicine, Juntendo University, Tokyo), Hiroyo Yoshino, BS (Research Institute for Diseases of Old Age, Graduate School of Medicine, Juntendo University, Tokyo) Yuanzhe Li, MD, PhD (Department of Neurology, Juntendo University School of Medicine, Tokyo), Yoko Imamichi (Department of Neurology, Juntendo University School of Medicine, Tokyo), Tatsushi Toda, MD (Division of Neurology/Molecular Brain Science, Kobe University Graduate School of Medicine, Kobe, Japan), Wataru Satake (Division of Neurology/Molecular Brain Science, Kobe University Graduate School of Medicine, Kobe, Japan). Funding: This work was supported by a grant from the Japanese Ministry of Education, Culture, Sports, Science and Technology, Grants-in-Aid for Scientific Research (to HT: 21591098, to NH: 090052131), for Scientific Research on Priority Areas (to NH: 08071510), and for Young Scientists (to MF: 22790829). *India:* Asha Kishore, MD (Sree Chitra Tirunal Institute for Medical Sciences and Technology, Trivandrum, Kerala, India) *Ireland:* Prof Tim Lynch FRCPI, FRCP (The Dublin Neurological Institute at the Mater Misericordiae University Hospital, Clinical Investigator at the Conway Institute, University College Dublin, Ireland) and Dr J. Mark Gibson, MD (Department of Neurology, Royal Victoria Hospital, Belfast, Ireland) *Italy:* Enza Maria Valente, MD, PhD (IRCCS, Casa Sollievo della Sofferenza Hospital, Mendel Institute, San Giovanni Rotondo), Alessandro Ferraris, MD, (IRCCS, Casa Sollievo della Sofferenza Hospital, Mendel Institute, San Giovanni Rotondo), Bruno Dallapiccola (Mendel Institute, Casa Sollievo della Sofferenza Hospital, Rome), Tamara Ialongo, MD, PhD, (Institute of Neurology, Catholic University, Rome), Laura Brighina MD PhD, (Department of Neurology, Ospedale San Gerarado, Monza, Italy), Barbara Corradi, PhD, (Department of Paediatrics, University of Milano-Bicocca, Monza), Roberto Piolti MD (Department of Neurology, Ospedale San Gerardo, Monza, Italy), Patrizia Tarantino, PhD (Institute of Neurological Sciences,National Research Council), Anna Rita Bentivoglio MD, PhD (Universita' Cattolica Del Sacro Cuore "Policlinico A. Gemelli", Rome, Italy), Carlo Ferrarese MD, PhD (Department of Neurology, San Gerardo Hospital, University of Milano-Bicocca, Monza, Italy) and Ferdinanda Annesi, PhD (Institute of Neurological Sciences, National Research Council). *Korea*: Beom S. Jeon, MD, PhD (Department of Neurology, Seoul National University Hospital), Sung-Sup Park, MD, PhD (Department of Laboratory Medicine, Seoul National University Hospital), Sun Ju Chung MD, PhD (Department of Neurology, Asan Medical Center, Seoul, Korea), Yun Joong Kim, MD (Hallym University, Anyang, Kyonggi-do, Korea) *Nigeria:* Njideka U. Okubadejo MD (University of Lagos, Lagos, Nigeria) *Norway:* J Aasly MD (Department of Neurology, University of Trondheim, Norway), *Peru*: Carlos Cosentino MD (Instituto de Ciencias Neurologicas, Lima, Peru) *Poland*: Grzegorz Opala, MD, PhD (Department of Neurology, Aging, Degenerative and Cerebrovascular Disorders, Medical University of Silesia, Katowice), Krygowska-Wajs MD, PhD (Department of Neurology, Collegium Medicum, Jagiellonian University, Krakow, Poland), Barbara Jasinska-Myga, MD, PhD (Department of Neurology, Aging, Degenerative and Cerebrovascular Disorders, Medical University of Silesia, Katowice), Gabriela Klodowska-Duda, MD, PhD (Department of Neurology, Aging, Degenerative and Cerebrovascular Disorders, Medical University of Silesia, Katowice), Magdalena Boczarska-Jedynak, MD, PhD (Department of Neurology, Aging, Degenerative and Cerebrovascular Disorders, Medical University of Silesia, Katowice) *Russia:* Sofya N. Pchelina PhD (Pavlov State Medical University of Saint Petersburg, Saint Petersburg, Russia), *Singapore:* Dr. Eng King Tan, MD, PhD (National Medical and Biomedical Research Councils, and the Duke-NUS Graduate

Medical School, Singapore Millenium Foundation), *South Africa:* Jonathan Carr, PhD (Division of Neurology, Department of Internal Medicine, University of Stellenbosch, Tygerberg, South Africa), *Spain:* Pao Pastor MD, PhD (Division of Neurosciences, Center for Applied Medical Research, University of Navarra School of Medicine, Pamplona, Spain) *Sweden:* Andrea Carmine Belin, PhD, (Department of Neuroscience, Karolinska Institutet, Stockholm), Lars Olson, Professor, (Department of Neuroscience, Karolinska Institutet, Stockholm), Dagmar Galter, PhD, (Department of Neuroscience, Karolinska Institutet, Stockholm), Marie Westerlund, PhD, (Department of Neuroscience, Karolinska Institutet, Stockholm), Karin Wirdefeldt MD, PhD (Karolinska Institutet, Stockholm, Sweden), Olof Sydow, PhD, (Department of Clinical Neuroscience, Karolinska University Hospital, Stockholm). Also Christer Nilsson, MD, PhD, (Department of Geriatric Psychiatry, Lund University), Andrea Puschmann, MD, (Department of Neurology, Lund University Hospital, Department of Geriatric Psychiatry, Lund University) *Taiwan:* JJ Lin, MD, Department of Neurology, Cushang Show-Chwan Hospital, Taiwan, *Turkey*: Ali Sazci PhD (University of Kocaeli, Kocaeli, Turkey), *UK:* Karen E. Morrison DPhil, University of Birmingham, UK, *USA*: Demetrius M. Maraganore, MD (Department of Neurology, NorthShore University HealthSystem, Evanston, IL, USA) J, Eric Ahlskog PhD, MD (Department of Neurology, Mayo Clinic, Rochester, MN, USA), Mariza de Andrade, PhD (Department of Health Sciences Research, Mayo Clinic, Rochester, MN, USA), Timothy G. Lesnick, MS (Department of Health Sciences Research, Mayo Clinic, Rochester, MN, USA), Tatiana Foroud, PhD (Indiana University, Indianapolis, IN, USA), Dimitri Krainc, MD, PhD (Massachusetts General Hospital, Harvard Medical School, Boston, USA) and Walter A. Rocca, MD, MPH (Departments of Neurology and Health Sciences Research, Mayo Clinic, Rochester, MN, USA), Beate Ritz MD, PhD (University of California, Los Angeles, CA, USA), John P. Ioannidis MD (Stanford University, Palo Alto, CA, USA). Also, Harvey Checkoway, PhD (Department of Environmental and Occupational Health Sciences, University of Washington, Seattle, WA), Owen A Ross PhD (Division of Neuroscience, Mayo Clinic, Jacksonville, USA), Zbigniew K. Wszolek, MD and Ryan J. Uitti, MD (Department of Neurology, Mayo Clinic, Jacksonville, FL). Funding: ZKW is partially supported by the NIH/NINDS 1RC2NS070276, NS057567, P50NS072187, Mayo Clinic Florida (MCF)Research Committee CR programs (MCF #90052018 and MCF #90052030), and the gift from Carl Edward Bolch, Jr., and Susan Bass Bolch (MCF #90052031/PAU #90052).

Supplementary References

1. Maraganore DM, de Andrade M, Lesnick TG, Strain KJ, Farrer MJ, et al. (2005) High-resolution whole-genome association study of Parkinson disease. Am J Hum Genet 77: 685–693. doi:10.1086/496902.

2. Dong H-rong, Hua Y, Ding X-sheng (2009) [Correlation study of semaphorin 5a with risk of Parkinson's disease in a Chinese Han population]. Zhonghua Yi Xue Za Zhi 89: 1589–1592.

3. He D, Guo J-feng, Wang L, Xiao Z-quan, Nie L-luo, et al. (2009) [Mutation analysis of ATP13A2 gene in Chinese patients with familial autosomal recessive early-onset parkinsonism]. Zhonghua Yi Xue Yi Chuan Xue Za Zhi 26: 567–570. doi:10.3760/cma.j.issn.1003-9406.2009.05.020.

4. Ding H, Wang F, Ding X, Song X, Lu X, et al. (2008) Association study of semaphorin 5A with risk of Parkinson's disease in a Chinese Han population. Brain Res 1245: 126–129. doi:10.1016/j.brainres.2008.09.080.

5. Guo J-F, Xiao B, Liao B, Zhang X-W, Nie L-L, et al. (2008) Mutation analysis of Parkin, PINK1, DJ-1 and ATP13A2 genes in Chinese patients with autosomal recessive early-onset Parkinsonism. Mov Disord 23: 2074–2079. doi:10.1002/mds.22156.

6. Larsson N-G (2010) Somatic mitochondrial DNA mutations in mammalian aging. Annu Rev Biochem 79: 683–706. doi:10.1146/annurev-biochem-060408-093701.

7. Purcell S, Neale B, Todd-Brown K, Thomas L, Ferreira MAR, et al. (2007) PLINK: a tool set for whole-genome association and population-based linkage analyses. Am J Hum Genet 81: 559–575. doi:10.1086/519795.

8. DerSimonian R, Laird N (1986) Meta-analysis in clinical trials. Control Clin Trials 7: 177–188.

9. Higgins JPT, Thompson SG (2002) Quantifying heterogeneity in a meta-analysis. Stat Med 21: 1539–1558. doi:10.1002/sim.1186.

10. Higgins JPT, Thompson SG, Deeks JJ, Altman DG (2003) Measuring inconsistency in meta-analyses. BMJ 327: 557–560. doi:10.1136/bmj.327.7414.557.

11. Ioannidis JPA, Patsopoulos NA, Evangelou E (2007) Uncertainty in heterogeneity estimates in meta-analyses. BMJ 335: 914–916. doi:10.1136/bmj.39343.408449.80.

12. Ioannidis JPA (2008) Effect of formal statistical significance on the credibility of observational associations. Am J Epidemiol 168: 374–383; discussion 384–390. doi:10.1093/aje/kwn156.

13. Wellcome Trust Case Control Consortium (2007) Genome-wide association study of 14,000 cases of seven common diseases and 3,000 shared controls. Nature 447: 661–678. doi:10.1038/nature05911.

14. Ioannidis JPA, Boffetta P, Little J, O'Brien TR, Uitterlinden AG, et al. (2008) Assessment of cumulative evidence on genetic associations: interim guidelines. Int J Epidemiol 37: 120–132. doi:10.1093/ije/dym159.

15. Khoury MJ, Bertram L, Boffetta P, Butterworth AS, Chanock SJ, et al. (2009) Genome-wide association studies, field synopses, and the development of the knowledge base on genetic variation and human diseases. Am J Epidemiol 170: 269–279. doi:10.1093/aje/kwp119.

16. Harbord RM, Egger M, Sterne JAC (2006) A modified test for small-study effects in meta-analyses of controlled trials with binary endpoints. Stat Med 25: 3443–3457. doi:10.1002/sim.2380.

17. Simón-Sánchez J, Schulte C, Bras JM, Sharma M, Gibbs JR, et al. (2009) Genome-wide association study reveals genetic risk underlying Parkinson's disease. Nat Genet 41: 1308–1312. doi:10.1038/ng.487.

18. Pankratz N, Wilk JB, Latourelle JC, DeStefano AL, Halter C, et al. (2009) Genomewide association study for susceptibility genes contributing to familial Parkinson disease. Hum Genet 124: 593–605. doi:10.1007/s00439-008-0582-9.

19. Marchini J, Howie B, Myers S, McVean G, Donnelly P (2007) A new multipoint method for genome-wide association studies by imputation of genotypes. Nat Genet 39: 906–913. doi:10.1038/ng2088.

20. Witte JS, Gauderman WJ, Thomas DC (1999) Asymptotic bias and efficiency in case-control studies of candidate genes and gene-environment interactions: basic family designs. Am J Epidemiol 149: 693–705.

21. Willer CJ, Li Y, Abecasis GR (2010) METAL: fast and efficient meta-analysis of genomewide association scans. Bioinformatics 26: 2190–2191. doi:10.1093/bioinformatics/btq340.

22. Nalls MA, Plagnol V, Hernandez DG, Sharma M, Sheerin U-M, et al. (2011) Imputation of sequence variants for identification of genetic risks for Parkinson's disease: a meta-analysis of genome-wide association studies. Lancet 377: 641–649. doi:10.1016/S0140-6736(10)62345-8.

23. Satake W, Nakabayashi Y, Mizuta I, Hirota Y, Ito C, et al. (2009) Genome-wide association study identifies common variants at four loci as genetic risk factors for Parkinson's disease. Nat Genet 41: 1303–1307. doi:10.1038/ng.485.

24. Sharma M, Ioannidis JPA, Aasly JO, Annesi G, Brice A, et al. (n.d.) Large-scale replication and heterogeneity in Parkinson disease genetic loci. Neurology in press.

25. Do CB, Tung JY, Dorfman E, Kiefer AK, Drabant EM, et al. (2011) Web-based genome-wide association study identifies two novel loci and a substantial genetic component for Parkinson's disease. PLoS Genet 7: e1002141. doi:10.1371/journal.pgen.1002141.

26. Spencer CCA, Plagnol V, Strange A, Gardner M, Paisan-Ruiz C, et al. (2011) Dissection of the genetics of Parkinson's disease identifies an additional association 5' of SNCA and multiple associated haplotypes at 17q21. Hum Mol Genet 20: 345–353. doi:10.1093/hmg/ddq469.

27. Hamza TH, Zabetian CP, Tenesa A, Laederach A, Montimurro J, et al. (2010) Common genetic variation in the HLA region is associated with late-onset sporadic Parkinson's disease. Nat Genet 42: 781–785. doi:10.1038/ng.642.

28. Saad M, Lesage S, Saint-Pierre A, Corvol J-C, Zelenika D, et al. (2011) Genome-wide association study confirms BST1 and suggests a locus on 12q24 as the risk loci for Parkinson's disease in the European population. Hum Mol Genet 20: 615–627. doi:10.1093/hmg/ddq497.

29. Simón-Sánchez J, van Hilten JJ, van de Warrenburg B, Post B, Berendse HW, et al. (2011) Genome-wide association study confirms extant PD risk loci among the Dutch. Eur J Hum Genet 19: 655–661. doi:10.1038/ejhg.2010.254.

30. Edwards TL, Scott WK, Almonte C, Burt A, Powell EH, et al. (2010) Genome-wide association study confirms SNPs in SNCA and the MAPT region as common risk factors for Parkinson disease. Ann Hum Genet 74: 97–109. doi:10.1111/j.1469-1809.2009.00560.x.

31. Bertram L, McQueen MB, Mullin K, Blacker D, Tanzi RE (2007) Systematic meta-analyses of Alzheimer disease genetic association studies: the AlzGene database. Nat Genet 39: 17–23. doi:10.1038/ng1934.

32. Allen NC, Bagade S, McQueen MB, Ioannidis JPA, Kavvoura FK, et al. (2008) Systematic meta-analyses and field synopsis of genetic association studies in schizophrenia: the SzGene database. Nat Genet 40: 827–834. doi:10.1038/ng.171.

33. Kent WJ, Zweig AS, Barber G, Hinrichs AS, Karolchik D (2010) BigWig and BigBed: enabling browsing of large distributed datasets. Bioinformatics 26: 2204–2207. doi:10.1093/bioinformatics/btq351.
